# A Unique Chimeric RNA: ERCC1‐iASPP Drives Benzo[a]pyrene‐Induced Lung Carcinogenesis via Dual Coding and Non‐Coding Mechanisms

**DOI:** 10.1002/advs.202507217

**Published:** 2025-11-26

**Authors:** Mingming Shan, Mingyang Xiao, Liang Zhang, Shen Wang, Jiahang Wu, Kang Cao, Yujin Li, Guopei Zhang, Junwei Xie, Xiaobo Lu

**Affiliations:** ^1^ Key Laboratory of Environmental Stress and Chronic Disease Control & Prevention Ministry of Education (China Medical University) Shenyang 110122 P. R. China; ^2^ Department of Toxicology School of Public Health China Medical University Shenyang 110122 P. R. China; ^3^ Department of Thoracic Surgery Liaoning Cancer Hospital & Institute Shenyang 110042 P. R. China

**Keywords:** bifunctional RNA, chimeric RNA, ERCC1‐iASPP, lung cancer, malignant transformation, polycyclic aromatic hydrocarbons

## Abstract

Genetic variation at 19q13.3 critically modulates chemical carcinogen‐induced lung carcinogenesis, particularly in mediating the activity of benzo[a]pyrene (B[a]P), a major polycyclic aromatic hydrocarbon (PAH) carcinogen. The adjacent genes *ERCC1* and *iASPP* within this locus respectively coordinate nucleotide excision repair of PAH‐induced DNA damage and suppression of apoptotic pathways. Their synergistic interaction regulates pivotal molecular events during PAH‐driven lung carcinogenesis, ultimately impacting cellular repair, proliferation, and apoptosis. Chimeric RNAs have been increasingly recognized as promising biomarkers and therapeutic targets in cancer. However, the characterization of lung cancer‐specific chimeric RNAs in the context of chemical carcinogenesis remains limited. This study identifies and characterizes *ERCC1‐iASPP*, a novel chimeric RNA derived from the neighboring genes *ERCC1* and *iASPP*, which exerts tumor‐promoting functions via coding and non‐coding mechanisms. First, the chimeric transcript encodes a previously uncharacterized protein, Ei, which enhances USP45‐mediated deubiquitination of ERCC1, thereby stabilizing ERCC1 protein. Additionally, *ERCC1‐iASPP* also functions as a (long non‐coding chimeric RNA, lnccRNA): in the cytoplasm, it acts as a (competing endogenous RNA, ceRNA) by sequestering miR‐143‐3p, leading to derepression of CDK1 and PGK1 and subsequent activation of oncogenic pathways, while in the nucleus, *ERCC1‐iASPP* further promotes transcriptional activation by recruiting STAT4 to the *PGK1* promoter. Collectively, these findings establish *ERCC1‐iASPP* as a bifunctional RNA with both protein‐coding and non‐coding regulatory roles that cooperatively promote B[a]P‐induced lung tumorigenesis. This study highlights *ERCC1‐iASPP* as a potential diagnostic and therapeutic target in smoking‐related lung cancer.

## Graphical abstract

The chimeric RNA *ERCC1‐iASPP* possesses dual coding and non‐coding functions, synergistically accelerating the process of cellular malignant transformation.

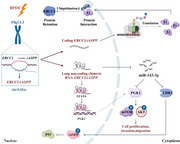

## 1 Introduction

Lung cancer, a significant global public health burden and leading cause of cancer death,^[^ ^1^ ^]^ is strongly linked to tobacco smoking.^[^ ^2^, ^3^ ^]^ The pathogenesis of this malignancy involves complex interactions between genetic predisposition and environmental exposures. Polycyclic aromatic hydrocarbons (PAHs), key carcinogenic constituents of tobacco smoke, contribute to lung carcinogenesis by promoting genomic instability—thus perpetuating a vicious cycle of PAHs exposure, genomic instability, gene mutation, and tumour development.^[^ ^4^, ^5^, ^6^ ^]^ Genomic instability can lead to structural rearrangements that drive the formation of oncogenic chimeric RNAs.^[^ ^7^, ^8^ ^]^ Notable examples include *EML4‐ALK*,^[^ ^9^, ^10^ ^]^ *FGFR3‐TACC3*,^[^ ^11^, ^12^ ^]^ and *KIF5B‐RET*,^[^ ^13^, ^14^ ^]^ which have been implicated in the malignant progression of lung cancer. Furthermore, replication stress or transcription‐replication conflicts arising from genomic instability can trigger aberrant transcriptional processes, generating non‐gene‐convergent chimeric RNAs via trans‐splicing or erroneous cis‐splicing events.^[^ ^15^, ^16^, ^17^, ^18^, ^19^ ^]^ While such mechanisms have been reported in other cancer types,^[^ ^20^, ^21^, ^22^, ^23^, ^24^, ^25^ ^]^ aberrant transcriptional regulation and splicing alterations induced by environmental carcinogens such as PAHs have yet to be reported in lung cancer.

Emerging evidence links genetic variations at chromosome 19q13.3 to altered lung cancer risk.^[^ ^26^, ^27^, ^28^ ^]^ Within this region, the *ERCC1* gene encodes a crucial component of the nucleotide excision repair (NER) pathway,^[^ ^29^, ^30^ ^]^ which removes bulky DNA adducts induced by carcinogens like PAHs.^[^ ^31^ ^]^ Adjacent to *ERCC1*, the *iASPP* oncogene is a negative regulator of p53‐mediated apoptosis.^[^ ^32^, ^33^ ^]^ Their frequent co‐overexpression in lung adenocarcinoma^[^ ^34^ ^]^ supports a synergistic “repair–apoptosis imbalance axis,” whereby enhanced DNA repair mediated by *ERCC1*,^[^ ^35^, ^36^ ^]^ coupled with *iASPP*‐mediated suppression of cell death,^[^ ^37^, ^38^ ^]^ promotes the survival of PAH‐damaged cells. Critically, preliminary sequencing data indicate the potential formation of a chimeric RNA involving these genes.^[^ ^39^ ^]^ The close genomic proximity (separated by only 975 bp) strongly implicates transcriptional read‐through—a mechanism increasingly implicated in oncogenesis—as the underlying process; this mechanism remains uninvestigated in the context of PAH exposure at this specific locus.

Over the past decade, increasing evidence has systematically illuminated the intricate landscape of RNA dysregulation in cancer.^[^ ^40^, ^41^ ^]^ These alterations affect both protein‐coding messenger RNAs (mRNAs)^[^ ^42^ ^]^ and a diverse array of non‐coding RNAs^[^ ^43^, ^44^, ^45^, ^46^ ^]^ that regulate essential cellular functions. Tumorigenesis is driven by the interplay between environmental exposures and intrinsic genetic susceptibility, with recent studies revealing that genotoxic stress—particularly from chemical carcinogens—can induce transcriptional read‐through,^[^ ^47^ ^]^ a process in which RNA polymerase II bypasses normal transcription termination signals and continues into adjacent downstream genes with convergent orientation. The resulting elongated transcripts are often processed through cis‐splicing of adjacent genes (cis‐SAGe), giving rise to chimeric RNAs composed of exons from two neighboring genes. These chimeric RNAs have emerged as novel oncogenic drivers, capable of promoting malignant transformation via gain‐of‐function mechanisms.^[^ ^48^, ^49^, ^50^, ^51^ ^]^ Notably, it is estimated that ≈4–5% of adjacent gene pairs in the human genome have the potential to generate such chimeric transcripts through the cis‐SAGe pathway,^[^ ^52^ ^]^ highlighting the widespread relevance of this transcriptional phenomenon in cancer biology.

Coding chimeric RNAs can generate fusion proteins with potent oncogenic activity, as exemplified by BCR‐ABL^[^ ^53^ ^]^ in chronic myeloid leukaemia (CML) and EML4‐ALK^[^ ^54^ ^]^ in non‐small cell lung cancer (NSCLC). In contrast, non‐coding chimeric RNAs^[^ ^55^ ^]^ contribute to tumorigenesis by disrupting cellular homeostasis through mechanisms such as miRNA sequestration—acting as ceRNAs—or by modulating chromatin remodelling. A recently recognized subclass, bifunctional chimeric RNAs, possesses both protein‐coding potential and non‐coding regulatory functions. These RNAs can regulate gene expression through non‐coding mechanisms, functioning as molecular decoys, scaffolds for protein or nucleic acid interactions, or signaling platforms that detect and respond to cellular metabolic states. Simultaneously, they retain the capacity to encode biologically active peptides or proteins via defined open reading frames (ORFs), such as the chimeric RNA SFT2D2‐TBX19^[^ ^56^ ^]^ in prostate cancer. From a therapeutic perspective, concurrently targeting both the protein products and the regulatory RNA functions of these molecules offers a synergistic strategy, potentially overcoming resistance mechanisms associated with single‐target therapies and advancing the development of precision oncology approaches.

In this study, we identified and characterized *ERCC1‐iASPP*, a novel chimeric RNA arising from transcriptional read‐through between the adjacent genes *ERCC1* and *iASPP* loci. We demonstrated that *ERCC1‐iASPP* functioned as a bifunctional transcript with temporally distinct roles during B[a]P‐induced genotoxic stress adaptation and lung carcinogenesis. Furthermore, its expression correlates with tobacco exposure and tumor stage in NSCLC patient cohorts, suggesting its clinical relevance as a biomarker and therapeutic target in smoking‐related lung cancer.

## 2 Results

### 2.1 Discovery of a Novel Chimeric RNA, *ERCC1‐iASPP*, in Lung Cancer

**Figure 1:**
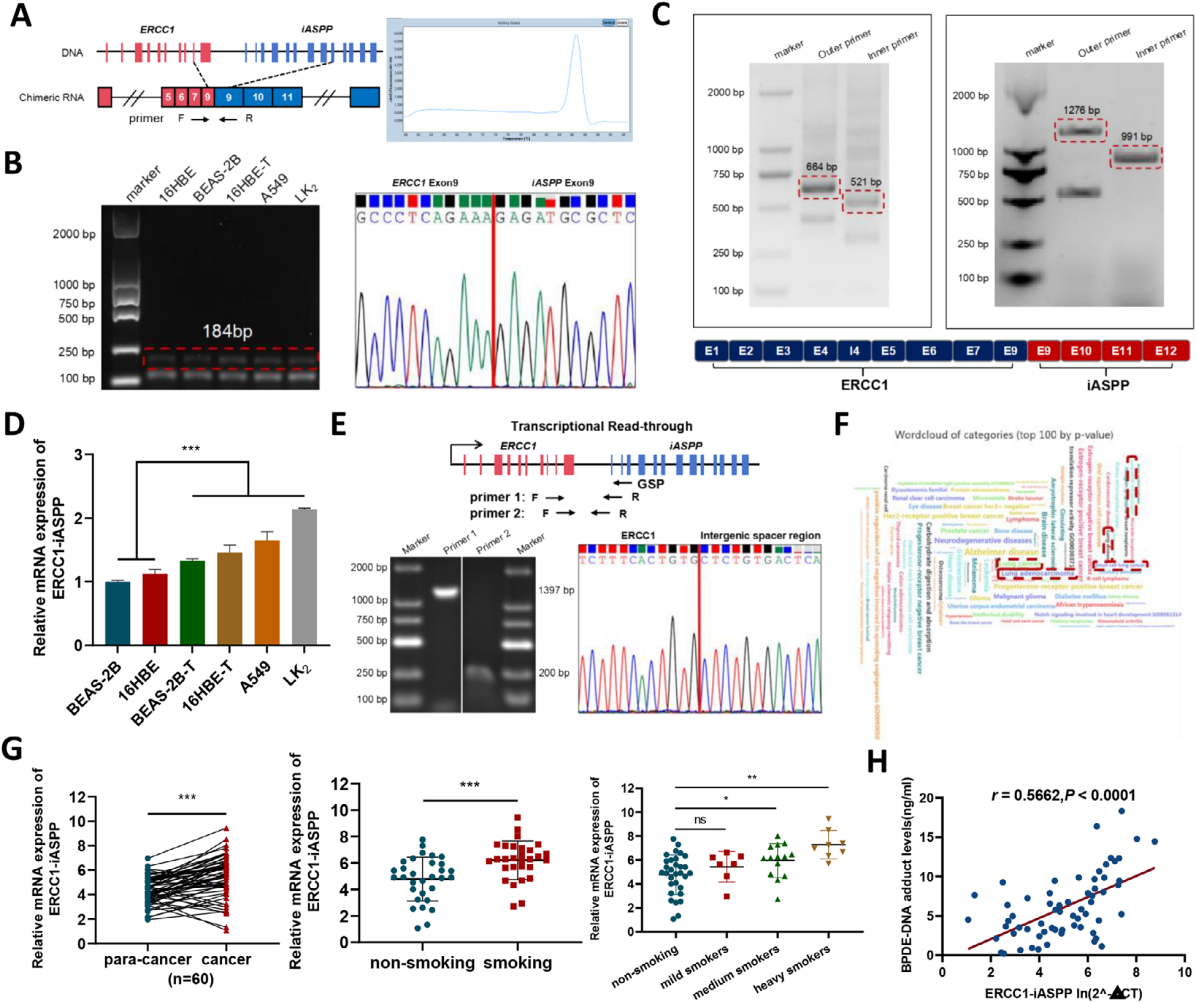
A novel oncogenic chimeric RNA *ERCC1‐iASPP* was found in lung cancer. A) Chimeric RNA *ERCC1‐iASPP* formation, schematic diagram of specific primer design, and peak melting curve. B) Agarose gel electrophoresis and Sanger sequencing determined the existence of *ERCC1‐iASPP*. C) 3'RACE, 5'RACE determined the full‐length sequence of *ERCC1‐iASPP*. D) mRNA expression of *ERCC1‐iASPP* in various cell lines, in which transformed malignant and lung cancer cells showed high expression levels. E) Primer design, PCR identification, and analysis of *ERCC1*, *iASPP* intergenic region. F) miEAA analysis of *ERCC1‐iASPP* disease‐associated cloud plots. G) *ERCC1‐iASPP* expression levels in lung cancer tissues and their adjacent nonmalignant tissues (n = 60), comparing the differential expression of *ERCC1‐iASPP* in cancer tissues of 29 smokers and 31 non‐smokers, and categorizing lung cancer cases into non‐smokers (n = 31), light smokers (n = 7), moderate smokers (n = 14), and heavy smokers (n = 8) according to the number of pack‐years of smoking, and comparing them with the non‐smokers. The smoking group was compared to analyze changes in *ERCC1‐iASPP* expression. Pack‐years = (packs smoked per day) × (years as a smoker). H) Correlation between BPDE‐DNA adduct concentration (ng/mL) and *ERCC1‐iASPP* mRNA expression in tissues of smokers (n = 32). ^*^ *P* < 0.05, ^**^ *P* < 0.01 and ^***^ *P* < 0.001. ns: the difference is not statistically significant. Data represent the mean ± SD. D: n = 3, Student's *t*‐test; G: Paired Samples *t*‐test and Kruskal‐Wallis test; H: Pearson Correlation Test.

According to Singh et al.^[^ ^39^ ^]^, *ERCC1‐iASPP* was predicted with distinct splicing junctions and an EricScore > 0.6 in prostate tissues, where it was consistently detected across three biological replicates, suggesting its plausible authenticity. Based on this bioinformatic evidence, we sought to experimentally verify the presence of *ERCC1‐iASPP* chimera using junction‐specific RT‐qPCR in two malignant lung cancer cell lines (A549 and LK_2_) and two transformed bronchial epithelial models (BEAS‐2B‐T and 16HBE‐T). Quantification analysis revealed a marked and specific overexpression of this previously uncharacterized chimeric transcript in malignant cells compared to normal pulmonary epithelia (**Figure** **1**A,B,D). To determine its full‐length structure, 5′‐ and 3′‐ rapid amplification of cDNA ends (RACE) was performed (Figure 1C) and Long‐range PCR (Figure S1B, Supporting Information), revealing a hybrid transcript comprising: It's composed of the exons 1–4 (with intron 4 retention), exons 5–7, and exon 9 of the parental *ERCC1* as well as exons 9–12 of the parental *iASPP*.

Chimeric RNAs can arise via gene fusion, trans‐splicing, or cis‐SAGe.^[^ ^57^ ^]^ Given the close genomic proximity between *ERCC1* and *iASPP*, we hypothesized that the cis‐SAGe was the underlying mechanism of *ERCC1‐iASPP* formation. No chimeric fragments were amplified when performing PCR using genomic DNA as a template (Figure S1A, Supporting Information). Furthermore, targeted RT‐qPCR amplification of the intergenic region followed by Sanger sequencing (Figure 1E) confirmed the presence of continuous transcription across both genes, consistent with a cis‐SAGe origin. The miEAA database further supported the oncogenic relevance of *ERCC1‐iASPP* (Figure 1F). Clinically, *ERCC1‐iASPP* expression could be detected in all cancers and matched adjacent tissues of non‐small cell lung cancer (NSCLC) (60/60). However, its expression levels were significantly lower in non‐malignant tissues versus tumors (*P* < 0.0001) (Figure 1G). Elevated transcript levels were significantly correlated with smoking history, lymphovascular invasion, and advanced TNM stages (Table S1, Supporting Information). Notably, smokers exhibited a 1.96 fold increase in tumor *ERCC1‐iASPP* expression compared to non‐smokers, with a dose‐dependent elevation observed in heavy smokers (Figure 1G). Intriguingly, expression levels positively correlated with BPDE‐DNA adduct accumulation—a biomarker of smoking‐induced genotoxic stress—in smoker‐derived tumors^[^ ^58^ ^]^ (Figure 1H). These data implicate *ERCC1‐iASPP* as a smoking‐associated molecular driver in the pathogenesis of NSCLC.

### 2.2 *ERCC1‐iASPP* Potentiates BPDE‐Induced Malignant Transformation and Amplifies Tumorigenic Phenotypes in Pulmonary Epithelial Cells

**Figure 2:**
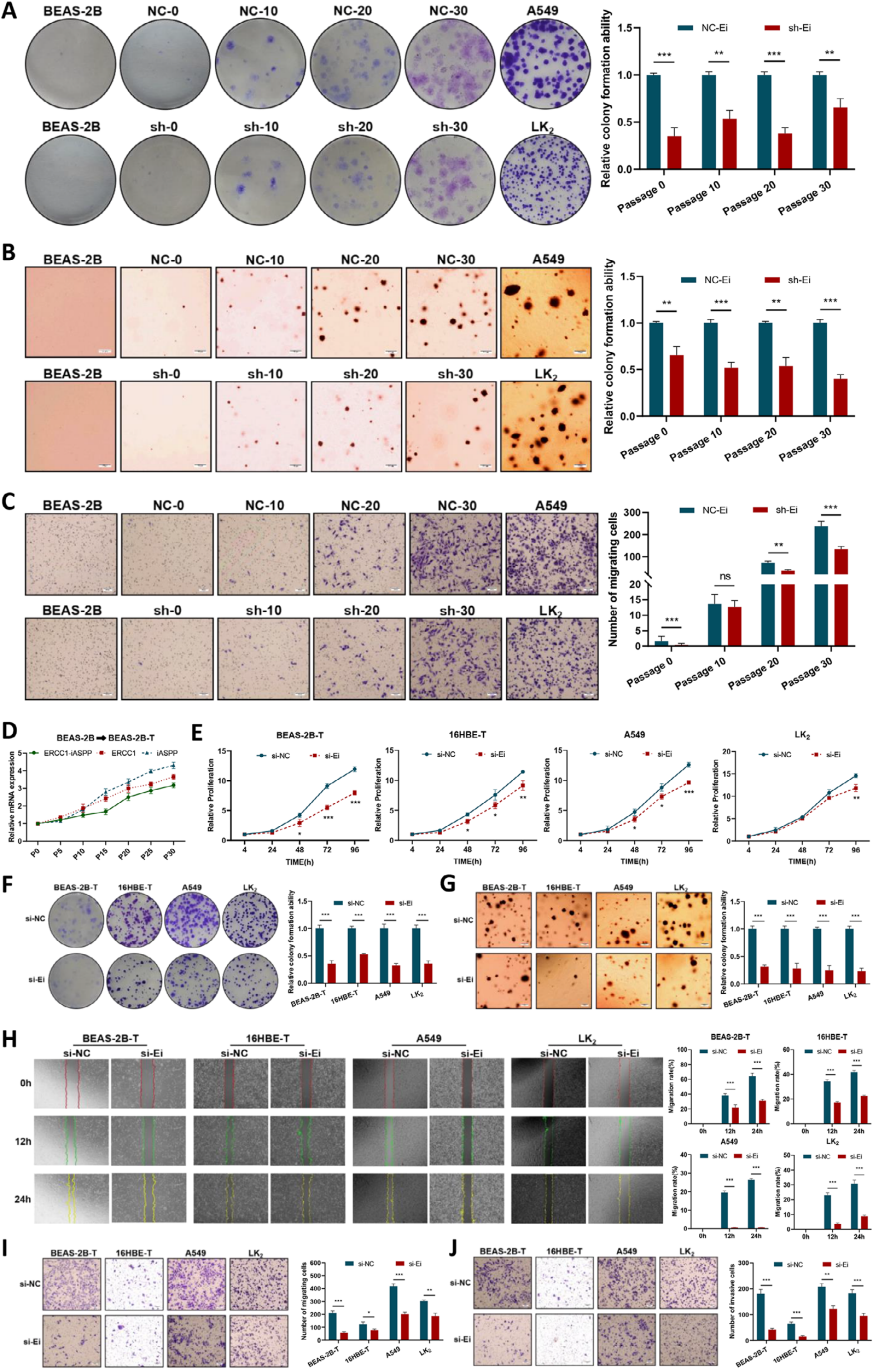
*ERCC1‐iASPP* accelerates the process of BPDE‐induced malignant transformation of lung epithelial cells and enhances the malignant phenotype of the cells. A–C) Stably transduced sh‐*ERCC1‐iASPP* and the negative control BEAS‐2B cells were induced with 0.5 µm BPDE (24 h each time, once a week for 4 consecutive weeks) respectively, and the plate colony formation, soft agar colony formation, and Transwell migration assay were performed to detect the malignancy degree of cells in generation 0, 10, 20, and 30. Scale bar = 500 µm. D) Changes in mRNA expression levels of chimeric RNA and parental genes with the number of generations of transformed cells during BPDE‐induced malignant transformation. E) to J) MTS E), plate colony formation F), soft agar colony formation G), wound healing H), and Transwell migration invasion assays I‐J) were used to evaluate the effects of *ERCC1‐iASPP* knockdown on the proliferation, colony formation, migration, and invasion abilities of each cell. Scale bar = 500 µm. ^*^ *P* < 0.05, ^**^ *P* < 0.01 and ^***^ *P* < 0.001. ns: differences were not statistically significant. Data represent the mean ± SD. D‐J: n = 3, Student's *t*‐test.

To investigate the functional role of *ERCC1‐iASPP* in BPDE‐induced carcinogenesis, stable knockdown models were established in bronchial epithelial cells (BEAS‐2B‐sh and 16HBE‐sh) using splice junction‐targeted shRNAs, suppressing chimeric RNA expression without altering the mRNA levels of the parental *ERCC1* or *iASPP* genes (Figure S2A, Supporting Information). Chronic exposure to BPDE (0.5 µm for BEAS‐2B; 1 µm for 16HBE) induced malignant transformation^[^ ^59^, ^60^, ^61^ ^]^ (Figure S2B, Supporting Information), as assessed across serial cell passages (BEAS‐2B: G0–G30; 16HBE: G0–G40). Functional assays revealed that *ERCC1‐iASPP* depletion significantly suppressed anchorage‐independent growth (**Figure** **2**A; Figure S2C, Supporting Information), cell proliferation (Figure 2B; Figure S2D, Supporting Information), and migration capacity (Figure 2C; Figure S2E, Supporting Information). Moreover, a dose‐dependent correlation between *ERCC1‐iASPP* expression and malignant progression, along with demonstrating its requirement for maintaining transformed phenotypes (Figure 2D; Figure S2F, Supporting Information), supports its role as a driver of BPDE‐mediated carcinogenesis. These findings align with clinical data linking *ERCC1‐iASPP* to smoking history and advanced TNM staging (Table S1, Supporting Information), suggesting its function in enhancing tolerance to genotoxic stress and promoting oncogenic transformation.

Further phenotypic assays, including MTS proliferation (Figure 2E), plate clone formation (Figure 2F), soft agar colony formation (Figure 2G), wound healing (Figure 2H), and Transwell‐based migration, and invasion (Figure 2I,J), demonstrated that knockdown of *ERCC1‐iASPP* (Figure S2G, Supporting Information) markedly inhibited the proliferative, clonogenic, migratory, and invasive capabilities of both stably transformed and malignant lung cancer cells. Collectively, these results implicate *ERCC1‐iASPP* as a novel oncogenic factor contributing to the developmental process of smoking‐type lung cancer.

### 2.3 *ERCC1‐iASPP*, as a Bifunctional Chimeric RNA, Accelerates the BPDE‐Induced Malignant Transformation Process

**Figure 3:**
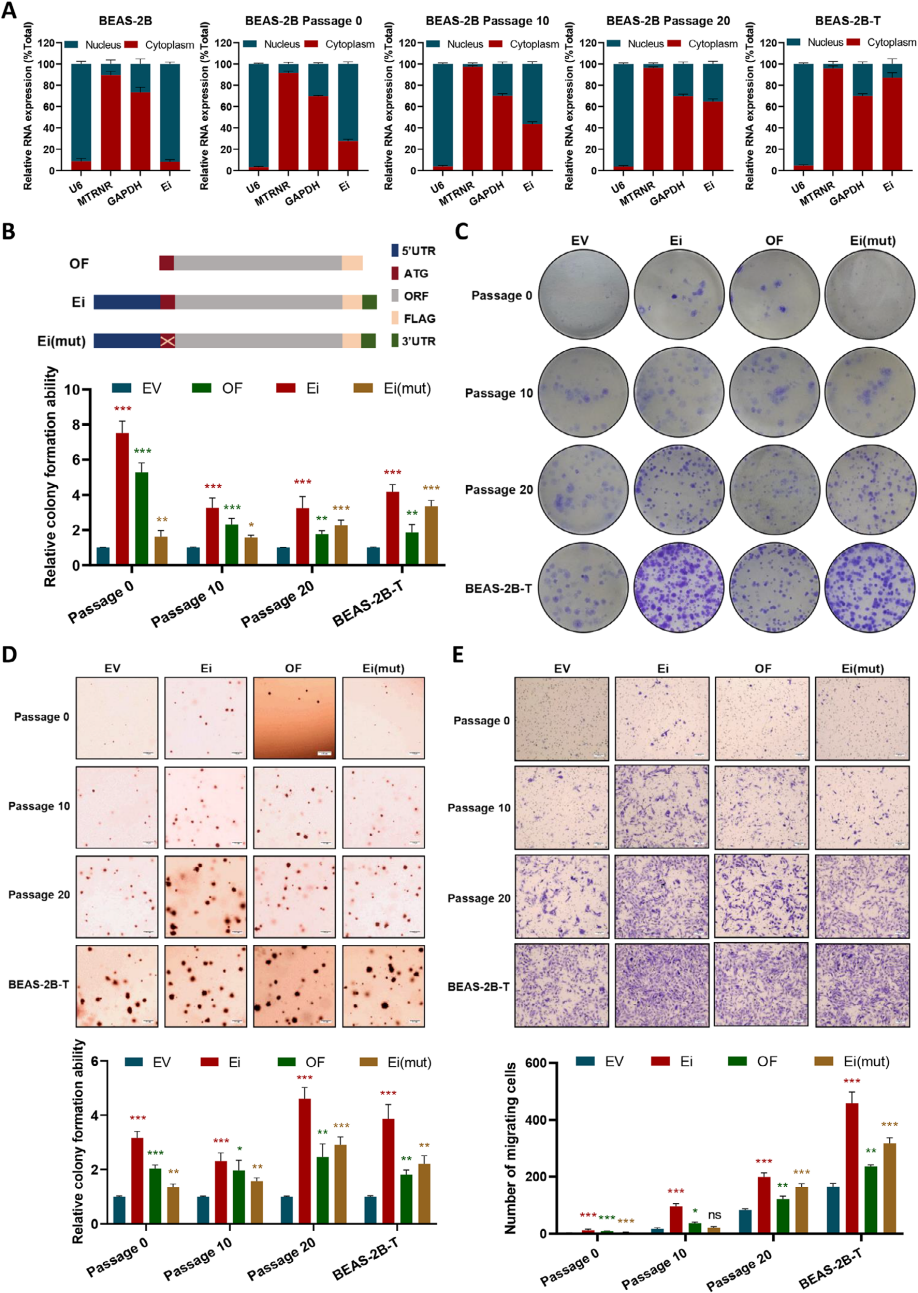
*ERCC1‐iASPP*, as a bifunctional chimeric RNA, combines coding and non‐coding dual functional properties to accelerate the BPDE‐induced malignant transformation process. A) RT‐qPCR analysis of *ERCC1‐iASPP* in nuclear and cytoplasm extracts, with *U6* used as a nucleus location reference and *MTRNR* as a cytoplasm location reference. With the enhancement of cell malignancy, *ERCC1‐iASPP* mRNA gradually shifted from nuclear distribution to cytoplasmic. B) Schematic diagram of the plasmid constructs. C–E) Representative images of plate colony formation, soft agar colony formation, and Transwell migration assay. Effect of plasmids with different properties of *ERCC1‐iASPP* on the anchorage‐independent colony formation ability of malrotransformed cells at each stage. ^*^ *P* < 0.05, ^**^ *P* < 0.01 and ^***^ *P* < 0.001. ns: differences were not statistically significant. Scale bar is 500 µm. Data represent the mean ± SD. A, C–E: n = 3, Student's *t*‐test.

The distinct subcellular localization pattern of the *ERCC1‐iASPP* chimeric RNA indicates its potential involvement in diverse regulatory processes. Dual nuclear‐cytoplasmic distribution was observed (**Figure** **3**A; Figure S3A, Supporting Information), indicating its capacity to engage in multiple molecular pathways. Notably, a progressive shift from cytoplasmic localization to nuclear retention was detected during increasing stages of cellular malignancy, implicating *ERCC1‐iASPP* in dynamic nucleocytoplasmic shuttling correlated with malignant progression. This observation prompted further investigation into its bifunctional roles—both as a protein‐coding RNA and as a long non‐coding chimeric RNA (lnccRNA). To dissect these mechanisms, three constructs were generated (Figure 3B; Figure S3B, Supporting Information): a) **OF**: a FLAG‐tagged open reading frame preserving translational capacity. b) **Ei**: a FLAG‐tagged full‐length sequence of *ERCC1‐iASPP*, which contains both coding and non‐coding functions. c) **Ei(mut)**: a Start codon‐mutated variant disabling protein synthesis while retaining RNA structure. These constructs were transfected into malignant cell models at varying transformation stages to assess the regulatory capacity of *ERCC1‐iASPP* via coding and non‐coding mechanisms.

Functional assays revealed that *ERCC1‐iASPP* expression significantly enhanced oncogenic phenotypes across all experimental conditions compared to vector controls (Figure 3C–E; Figure S3C–E, Supporting Information). The Ei construct, containing both functional modalities, demonstrated the most pronounced oncogenic activity. Notably, the OF construct (protein‐coding only) showed stronger effects during early‐stage transformation, whereas the Ei(mut) construct (lnccRNA‐only) conferred greater oncogenic potential during advanced stages. These results establish *ERCC1‐iASPP* as a bifunctional chimera RNA that not only encodes a malignancy‐associated protein but also retains non‐coding regulatory capacity, both of which contribute synergistically to BPDE‐induced lung tumorigenesis through context‐dependent and stage‐specific mechanisms.

### 2.4 *ERCC1‐iASPP* Encodes a Novel Protein and Correlates with ERCC1 Protein Expression Level

**Figure 4:**
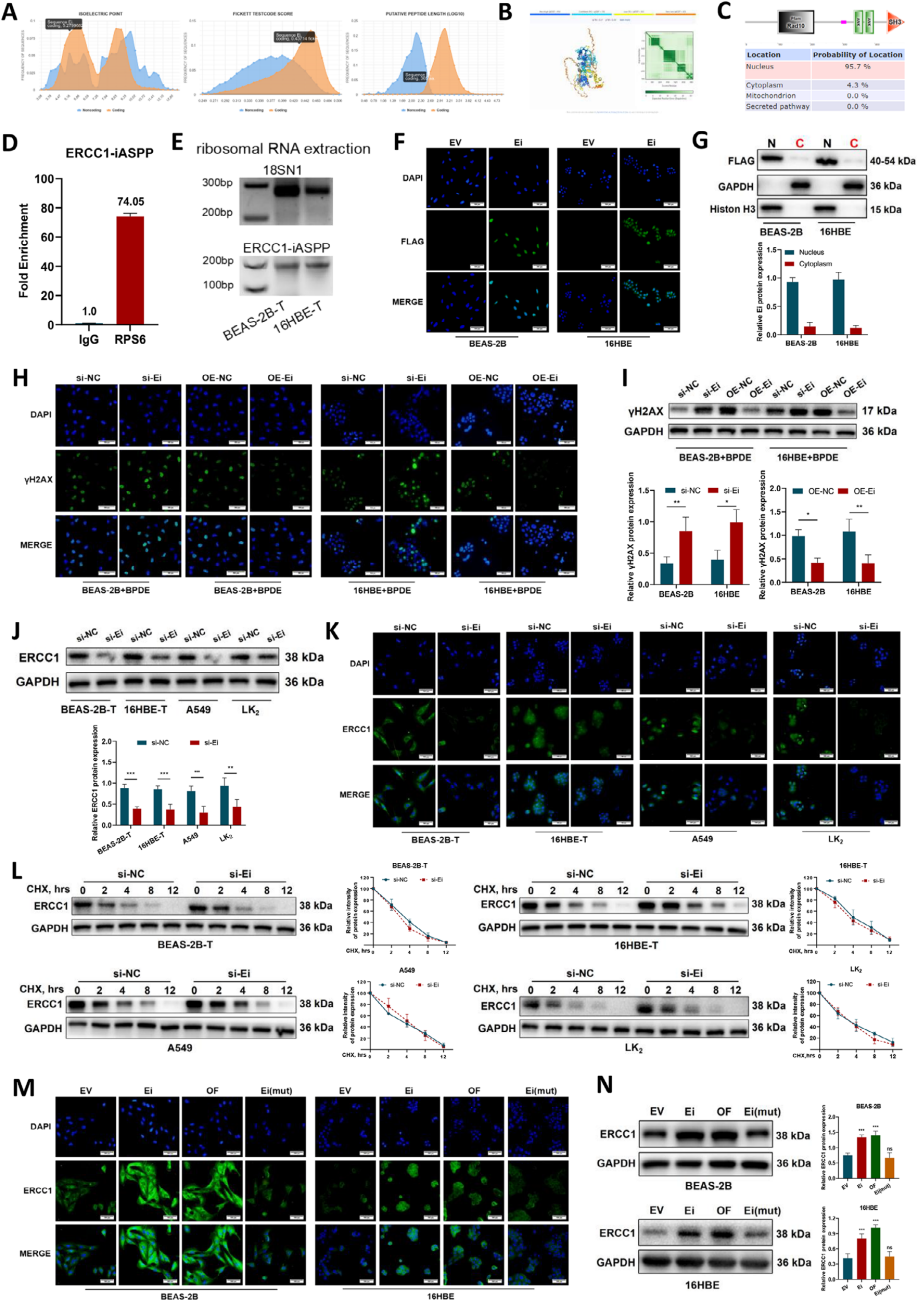
Prediction and validation of *ERCC1‐iASPP* encoding capacity and the effect of the novel protein Ei on ERCC1 protein. A–C) The CPC2 database predicted the encoding ability of *ERCC1‐iASPP*, the AlphaFold3 predicted the tertiary structure of the new protein, and the SMART and YLoc databases predicted the functional structural domain composition and subcellular localization of the new protein. D) RIP experiments using primers specific for *ERCC1‐iASPP* revealed a fold enrichment of *ERCC1‐iASPP* by anti‐RPS6 antibody. E) rRNA was extracted for PCR amplification, and the products were detected by agarose gel electrophoresis for *ERCC1‐iASPP* expression. 18S rRNA was utilized as a positive control. F) Novel protein expression of *ERCC1‐iASPP* was detected by immunofluorescence assay using a FLAG‐tagged antibody. G) The new ERCC1‐iASPP protein is predominantly localized in the nucleus. H,I) Changes in the expression of the cellular DNA damage marker γH2AX were detected by immunofluorescence H) and Western blot I) after altering *ERCC1‐iASPP* expression. J–L) Western blot J), immunofluorescence K), and actinomycin D chase assays L) were used to detect the changes of ERCC1 protein expression, subcellular localization, and degradation rate upon the reduction of *ERCC1‐iASPP* expression. M,N) Immunofluorescence M) and Western blot N) clarified that the effect of ERCC1‐iASPP on ERCC1 protein depends on its encoding ability. ^*^ *P* < 0.05, ^**^ *P* < 0.01 and ^***^ *P* < 0.001. ns: differences were not statistically significant. Scale bar is 500 µm. Data represent the mean ± SD. D, G, I, J, L: n = 3, Student's *t*‐test. N: n = 3, one‐way ANOVA with Fisher's LSD.

Bioinformatic analyses using ORF Finder and CPC2 identified a conserved open reading frame (ORF) within *ERCC1‐iASPP*, indicating a high coding probability (**Figure** **4**A). Domain architecture prediction via SMART/YLoc revealed a chimeric protein comprising a partial RAD10 DNA repair domain from *ERCC1* fused with the SH3 protein interaction module from *iASPP* (Figure 4B, C), with a predicted preference for nuclear localization. To assess translational potential, RNA immunoprecipitation using anti‐RPS6 antibodies—a ribosomal protein associated with active translation^[^ ^62^ ^]^—demonstrated a 74.05 fold enrichment of *ERCC1‐iASPP* compared to IgG controls (Figure 4D). The profiling further confirmed its association with translational machinery, as evidenced by detectable amplification of *ERCC1‐iASPP* from ribosome‐bound fractions (Figure 4E). On the other hand, to validate translational capacity, the Ei construct was transfected into BEAS‐2B and 16HBE cells. Immunofluorescence using anti‐FLAG antibodies confirmed specific signals exclusively in Ei‐transfected cells (Figure 4F). Subcellular fractionation and Western blotting confirmed Flag‐tagged protein expression at the predicted molecular weights, with predominant nuclear localization (Figure 4G). Collectively, these results suggest that *ERCC1‐iASPP* encodes a novel, nucleus‐enriched chimeric protein.

Although BPDE primarily exerts its carcinogenic effects through DNA damage,^[^ ^63^ ^]^ the functional implications of BPDE‐induced chimeric transcripts remain unclear. In this study, we investigated the impact of the chimeric transcript *ERCC1‐iASPP* on the repair capacity of BPDE‐induced DNA damage. Notably, despite lacking exon 8 of the parental *ERCC1* gene‐a region essential for canonical DNA repair ‐ overexpression of *ERCC1‐iASPP* (Figure S4A, Supporting Information) significantly reduced BPDE‐induced DNA damage (Figure 4H,I). RT‐qPCR confirmed that *ERCC1‐iASPP* manipulation did not affect endogenous *ERCC1* mRNA levels (Figure S4A, Supporting Information), excluding the mechanism of transcriptional regulation. Protein‐level analyses—including Western blot (Figure 4J), immunofluorescence (Figure 4K), and actinomycin D chase assays (Figure 4L)—demonstrated that *ERCC1‐iASPP* may enhance ERCC1 protein stability without affecting its localization or degradation kinetics.

Given the bifunctional nature of *ERCC1‐iASPP* as a chimeric RNA with dual coding and non‐coding potential, we systematically dissected its functional domains to identify the mechanism underlying ERCC1 protein regulation. To further elucidate the mechanism, BEAS‐2B and 16HBE cells were transfected with three distinct constructs (Ei, OF, Ei(mut)) (Figure S3B, Supporting Information). Both Immunofluorescence (Figure 4M) and Western blot analyses (Figure 4N) revealed that ERCC1 protein levels were significantly elevated in cells expressing Ei or OF, while this effect was abolished in the non‐coding Ei(mut) construct. These findings collectively demonstrate that the ability of *ERCC1‐iASPP* to regulate ERCC1 protein expression is dependent on its intrinsic coding capacity (Figure S4B, Supporting Information), confirming the functional role of the encoded chimeric protein in modulating DNA damage response.

### 2.5 Novel Protein Ei (Encoded by *ERCC1‐iASPP*) Reduces ERCC1 Ubiquitination by Recruiting USP45

**Figure 5:**
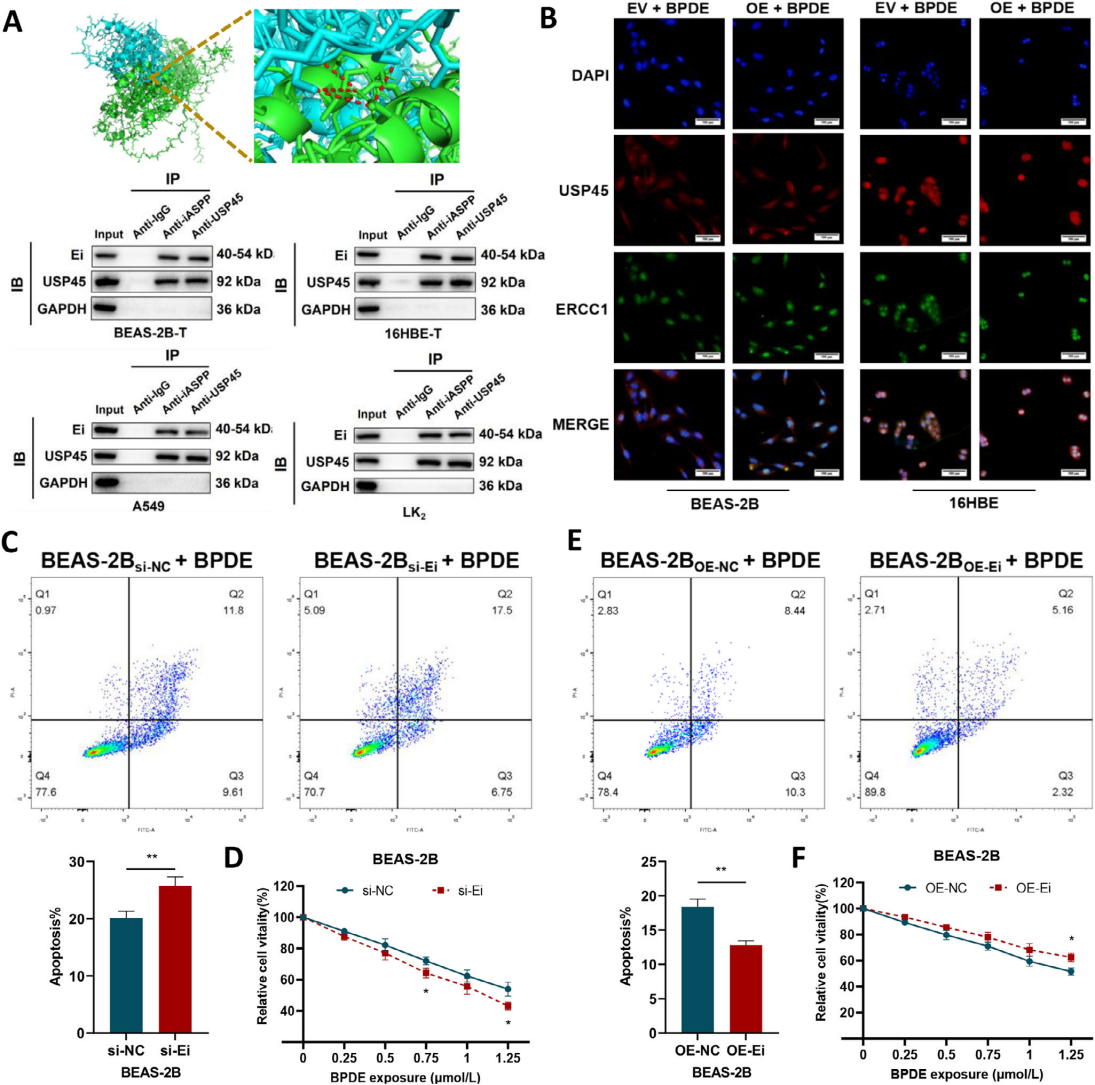
Recruitment of USP45 by Ei decreases the level of ubiquitination of the ERCC1 protein. This inhibits BPDE‐induced apoptosis and increases cellular activity. A) PyMOL prediction and Co‐IP validation of direct protein‐protein interactions between the novel protein Ei and USP45. B) Immunofluorescence detection of Ei recruits USP45 to the nucleus to better reduce the level of ubiquitination of the ERCC1 protein. C,D) Flow cytometry C) and MTS assay D) were performed to detect the effects of *ERCC1‐iASPP* knockdown on BPDE‐induced apoptosis and cell metabolic activity in BEAS‐2B cells. E,F) The effects of *ERCC1‐iASPP* overexpression on BPDE‐induced apoptosis and cell activity in BEAS‐2B cells were determined by flow cytometry E) and MTS assay F). ^*^ *P* < 0.05, ^**^ *P* < 0.01 and ^***^ *P* < 0.001. ns: differences were not statistically significant. Scale bar is 500 µm. Data represent the mean ± SD. C–F: n = 3, Student's *t*‐test.

To elucidate the underlying molecular mechanism, we focused on USP45,^[^ ^64^ ^]^ a deubiquitinating enzyme known to regulate ERCC1 stability via reversible ubiquitination. Protein–protein interaction prediction algorithms, along with Co‐IP assays, confirmed a direct binding between the chimeric Ei protein encoded by *ERCC1‐iASPP* and endogenous USP45 (**Figure** **5**A). Notably, BPDE exposure enhanced nuclear translocation of the Ei‐USP45 complex (Figure 5B), facilitating its interaction with ERCC1 and resulting in a marked reduction in ERCC1 ubiquitination. This dynamic relocalization suggests a stress‐responsive mechanism, wherein the Ei protein functions as a molecular scaffold that recruits USP45 to stabilize ERCC1 through targeted deubiquitination. Consistently, knockdown of *ERCC1‐iASPP* in BPDE‐treated BEAS‐2B/16HBE cells significantly increased cytotoxicity, as evidenced by increased apoptosis levels (Figure 5C; Figure S4D, Supporting Information) and reduced metabolic activity (Figure 5D; Figure S4E, Supporting Information). In contrast, overexpression of *ERCC1‐iASPP* conferred protection against BPDE‐induced stress, decreasing apoptosis and enhancing cell viability (Figure 5E,F; Figure S4F,G, Supporting Information). These data demonstrate that *ERCC1‐iASPP* encodes the Ei protein and plays a critical role in mitigating genotoxic stress by promoting ERCC1 stability, thereby enabling cellular survival under BPDE‐induced DNA damage.

### 2.6 LnccRNA *ERCC1‐iASPP* Functions as a Cytoplasmic Molecular Sponge by Sequestering miR‐143‐3p

**Figure 6:**
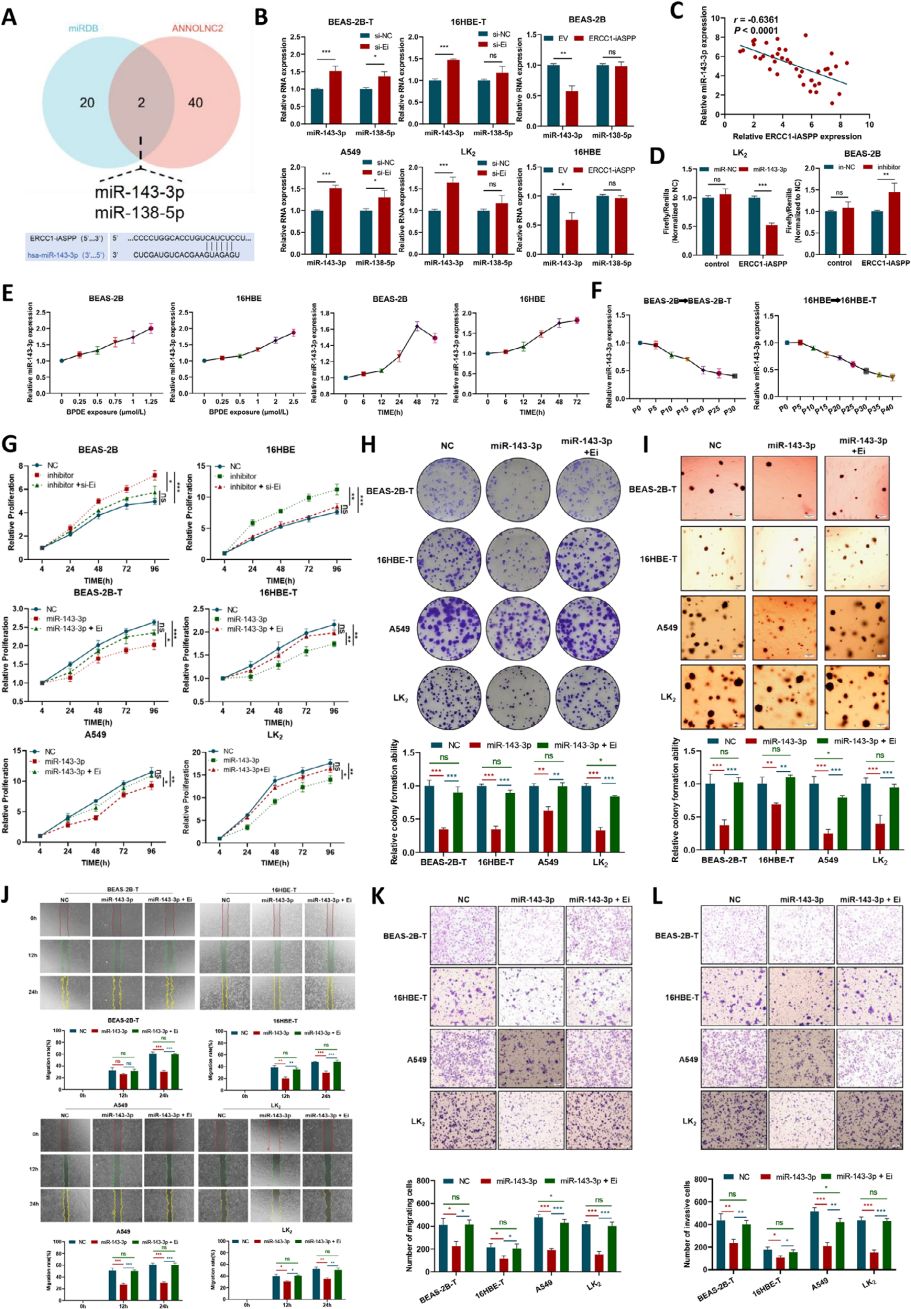
The long non‐coding chimeric RNA *ERCC1‐iASPP* in the cytoplasm sequesters and represses miR‐143‐3p to enhance the malignant phenotype of the cells. A,B) Screening of miRNAs by databases and experiments. C) The correlation of the expression of miR‐143‐3p with that of *ERCC1‐iASPP* in lung carcinoma and paracancerous tissues (n=40). D) Dual luciferase reporter assay showed the interaction of miR‐143‐3p and *ERCC1‐iASPP*. E) Dose‐effect and time‐effect relationship of miR‐143‐3p expression after BPDE exposure. F) BPDE induced cells to undergo the malignant transformation process. MiR‐143‐3p expression levels with the number of generations of transformed cells. G–L) The effects of miR‐143‐3p mimic and overexpression of *ERCC1‐iASPP* on the degree of malignancy of malignant transformation and lung cancer cells were assessed by the MTS G), plate clone formation H), soft agar colony formation I), wound healing J), Transwell migration and invasion K), and L) assays. ^*^ *P* < 0.05, ^**^ *P* < 0.01 and ^***^ *P* < 0.001. ns: differences were not statistically significant. Scale bar is 500 µm. Data represent the mean ± SD. B, D: n = 3, Student's *t*‐test. C: Pearson Correlation Test. G–L: n = 3, one‐way ANOVA with Fisher's LSD.

Functional characterization of *ERCC1‐iASPP* as a lnccRNA reveals the mechanistic similarities to conventional lncRNAs. Its predominant cytoplasmic localization supports a potential role as a competing ceRNA^[^ ^65^ ^]^ that regulates gene expression through microRNA sequestration. Bioinformatics analyses using miRDB and ANNOLNC2 platforms identified two candidate miRNA binding partners (**Figure** **6**A; Figure S5A,B, Supporting Information). Subsequent experimental validation demonstrated that miR‐143‐3p, but not miR‐138‐5p, exhibited a reciprocal expression pattern in response to *ERCC1‐iASPP* modulation: miR‐143‐3p levels were downregulated following *ERCC1‐iASPP* overexpression and upregulated upon its silencing (Figure 6B). This inverse relationship was further confirmed in human lung tumor specimens, where a significant negative correlation between *ERCC1‐iASPP* and miR‐143‐3p expression was observed (Figure 6C). Dual‐luciferase reporter assays demonstrated that miR‐143‐3p directly binds to *ERCC1‐iASPP*, as its overexpression significantly suppressed reporter activity, whereas miR‐143‐3p inhibition enhanced it (Figure 6D; Figure S5C,D, Supporting Information).

Notably, miR‐143‐3p exhibited a biphasic expression pattern during BPDE‐induced carcinogenesis: it was transiently upregulated following short‐term BPDE exposure (Figure 6E), but progressively downregulated during long‐term malignant transformation (Figure 6F). These findings suggest a context‐dependent functional duality, wherein *ERCC1‐iASPP* may preferentially exert coding functions during acute stress and non‐coding regulatory roles during chronic oncogenic progression.

To functionally validate this regulatory axis, rescue experiments were performed (Figure S5E,F, Supporting Information). Overexpression of miR‐143‐3p mimic significantly suppressed malignant phenotypes, including reduced proliferation (Figure 6G), clonogenic potential (Figure 6H,I), and invasive capacity (Figure 6J–L). Crucially, co‐overexpression of *ERCC1‐iASPP* with miR‐143‐3p reversed these tumor‐suppressive effects, demonstrating the ability of *ERCC1‐iASPP* to antagonize miR‐143‐3p and promote oncogenic progression.

### 2.7 Prediction and Validation of Target Genes Involved in *ERCC1‐iASPP*/miR‐143‐3p Axis

**Figure 7:**
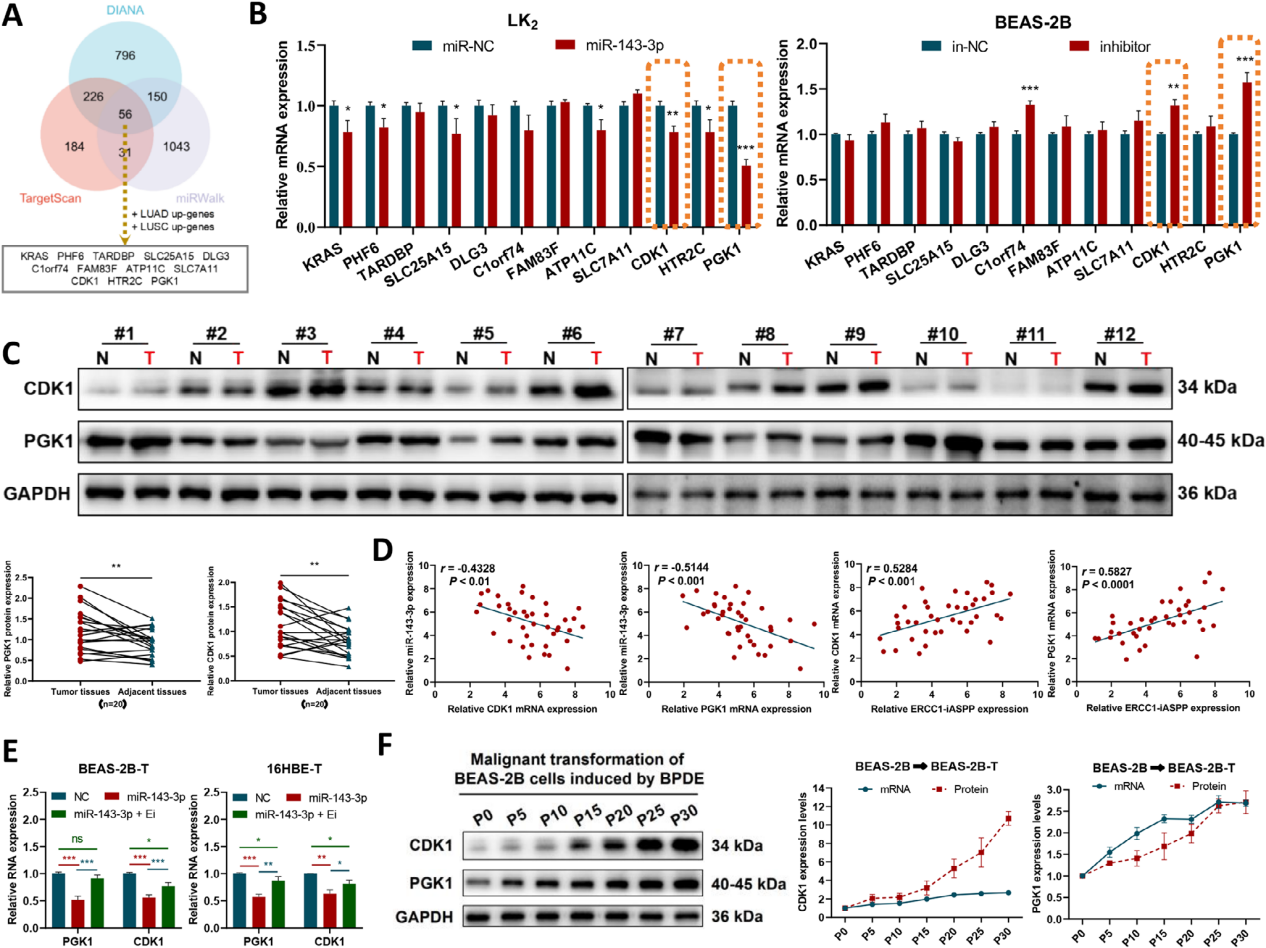
Prediction and validation of *ERCC1‐iASPP*/miR‐143‐3p axis target genes. A,B) Screening of target genes by databases and experiments. C) Differential expression of the target genes, CDK1 and PGK1 proteins, in lung cancers and paired paracancerous tissues. D) The correlation between *CDK1* and *PGK1* mRNA and miR‐143‐3p in lung cancers and paired paracancerous tissues, as well as *ERCC1‐iASPP* expression. E) RT‐qPCR detection of miR‐143‐3p mimic and altered *CDK1*, *PGK1* mRNA expression after overexpression of *ERCC1‐iASPP*. F) CDK1, PGK1 mRNA, and protein expression during BPDE‐induced malignant transformation of BEAS‐2B cells. ^*^ *P* < 0.05, ^**^ *P* < 0.01 and ^***^ *P* < 0.001. ns: differences were not statistically significant. Scale bar is 500 µm. Data represent the mean ± SD. B: n = 3, Student's t test. C: Paired Samples *t*‐test. D: Pearson Correlation Test. E: n = 3, one‐way ANOVA with Fisher's LSD.

MiR‐143‐3p remains relatively underexplored in oncogenic contexts. To identify its potential downstream targets, an intersectional analysis was conducted using three bioinformatics platforms—DIANA, TargetScan, and miRWalk—combined with transcriptomic data from lung adenosquamous carcinoma. This intersectional analysis yielded 12 candidate target genes (**Figure** **7**A). Among them, RT‐qPCR validation revealed *CDK1* and *PGK1* as the only genes exhibiting an inverse correlation with miR‐143‐3p expression (Figure 7B). Consistently, analysis of clinical specimens demonstrated significant upregulation of CDK1 and PGK1 protein levels in tumor tissues compared to paired adjacent normal tissues (Figure 7C; Figure S6A, Supporting Information). Correlation analysis further showed a strong negative association between miR‐143‐3p and both *CDK1/PGK1*, and a positive correlation with *ERCC1‐iASPP* across tumor samples (Figure 7D; Figure S6B, Supporting Information).

Functional studies confirmed that overexpression of miR‐143‐3p suppressed the expression of *CDK1*/*PGK1*, while co‐overexpression with *ERCC1‐iASPP* rescued their suppression (Figure 7E; Figure S6C, Supporting Information), supporting a ceRNA‐mediated regulatory mechanism. Clinically, elevated *CDK1*/*PGK1* expression levels were significantly associated with smoking history in lung cancer patients (Figure S6D, Supporting Information). Moreover, longitudinal analysis of BPDE‐induced cellular transformation models revealed a stepwise increase in *CDK1/PGK1* mRNA and protein levels that paralleled malignant progression (Figure 7F; Figure S6E, Supporting Information).

### 2.8 *ERCC1‐iASPP* Co‐Targets CDK1 and PGK1 by miR‐143‐3p to Promote BPDE‐Induced Malignant Transformation

**Figure 8:**
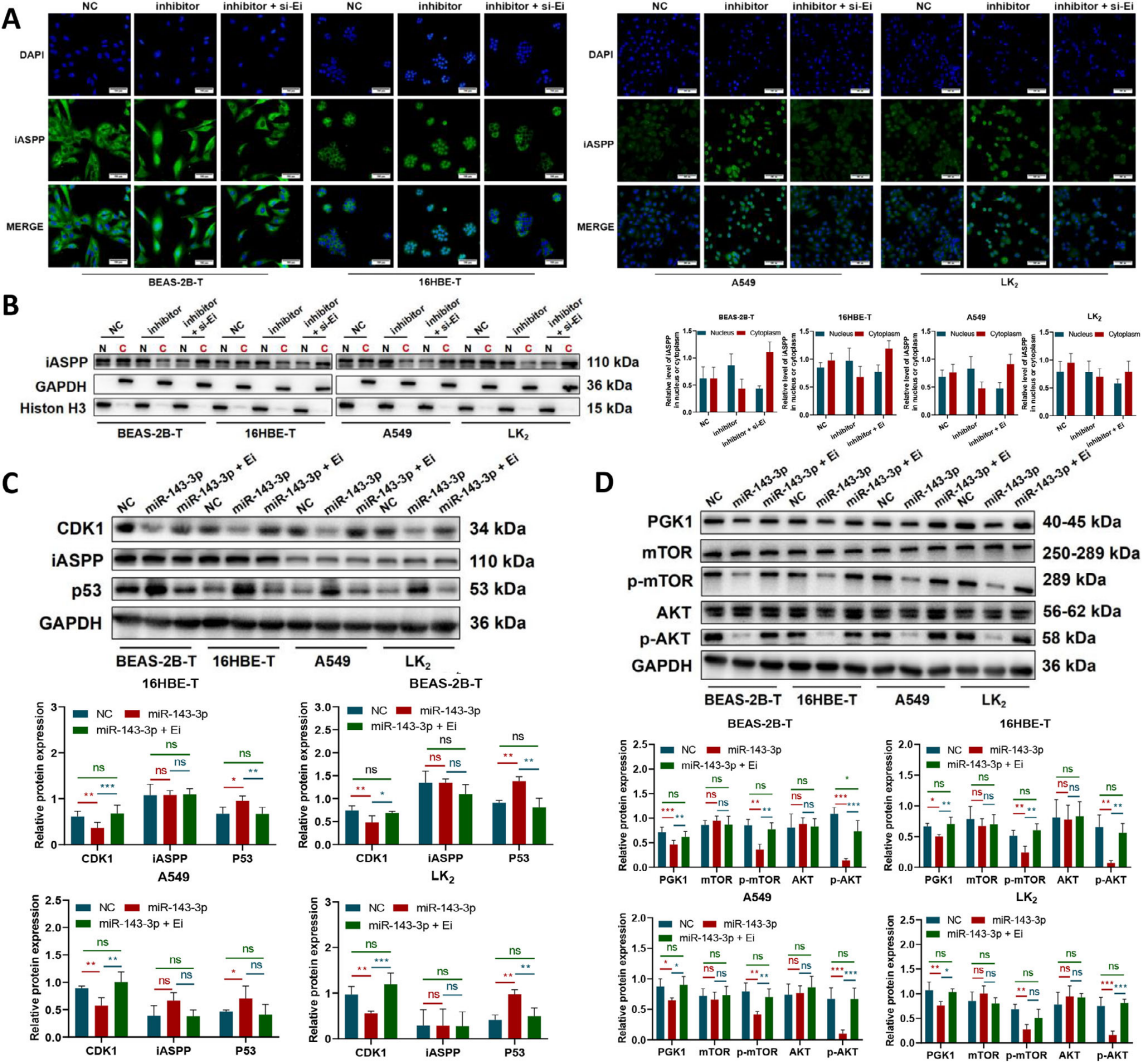
*ERCC1‐iASPP* co‐targets miR‐143‐3p/CDK1, miR‐143‐3p/PGK1 axis to accelerate BPDE‐induced cellular malignancy. A,B) Immunofluorescence A) and cytosolic protein extraction procedure B) were employed to detect changes in the distribution of iASPP proteins between nuclear and cytoplasmic compartments after alterations in the expression of *ERCC1‐iASPP* and miR‐143‐3p. C,D) Western blot analysis of the alterations in *ERCC1‐iASPP* and miR‐143‐3p expression detected alterations in CDK1 and PGK1, along with molecular protein changes associated with their respective downstream pathways. ^*^ *P* < 0.05, ^**^ *P* < 0.01 and ^***^ *P* < 0.001. ns: differences were not statistically significant. Scale bar is 500 µm. Data represent the mean ± SD. C, D: n = 3, one‐way ANOVA with Fisher's LSD.

MiR‐143‐3p is known to attenuate CDK1‐mediated phosphorylation of iASPP, a post‐translational modification critical for its nuclear translocation and subsequent interaction with p53.^[^ ^66^, ^67^ ^]^ In this study, inhibition of miR‐143‐3p led to increased nuclear fluorescence intensity of iASPP (**Figure** **8**A) and elevated nuclear protein levels (Figure 8B). These effects were completely reversed upon co‐treatment with miR‐143‐3p inhibitor and *ERCC1‐iASPP*‐targeting siRNA, confirming the regulatory role of *ERCC1‐iASPP* in this axis. Additionally, miR‐143‐3p‐induced upregulation of p53 was abolished by co‐overexpression of *ERCC1‐iASPP* (Figure 8C), suggesting that *ERCC1‐iASPP* suppresses p53 activation via modulation of CDK1/iASPP signaling. Parallel analyses identified PGK1 as a functional mediator of the mTOR/AKT pathway, which plays a critical role in oncogenic signaling. Overexpression of miR‐143‐3p significantly reduced PGK1 protein expression, accompanied by decreased phosphorylation of mTOR and AKT.^[^ ^68^ ^]^ Notably, co‐overexpression of *ERCC1‐iASPP* restored PGK1 levels and rescued mTOR/AKT phosphorylation (Figure 8D), confirming that *ERCC1‐iASPP* antagonizes miR‐143‐3p‐mediated suppression of key oncogenic signaling pathways. These findings collectively demonstrate that *ERCC1‐iASPP* promotes BPDE‐induced malignant transformation through coordinated regulation of the miR‐143‐3p/CDK1 and miR‐143‐3p/PGK1 axes.

### 2.9 Nuclear‐Enriched lnccRNA *ERCC1‐iASPP* Recruits STAT4 at *PGK1* Promoter to Form a Transcriptional Activation Complex

**Figure 9:**
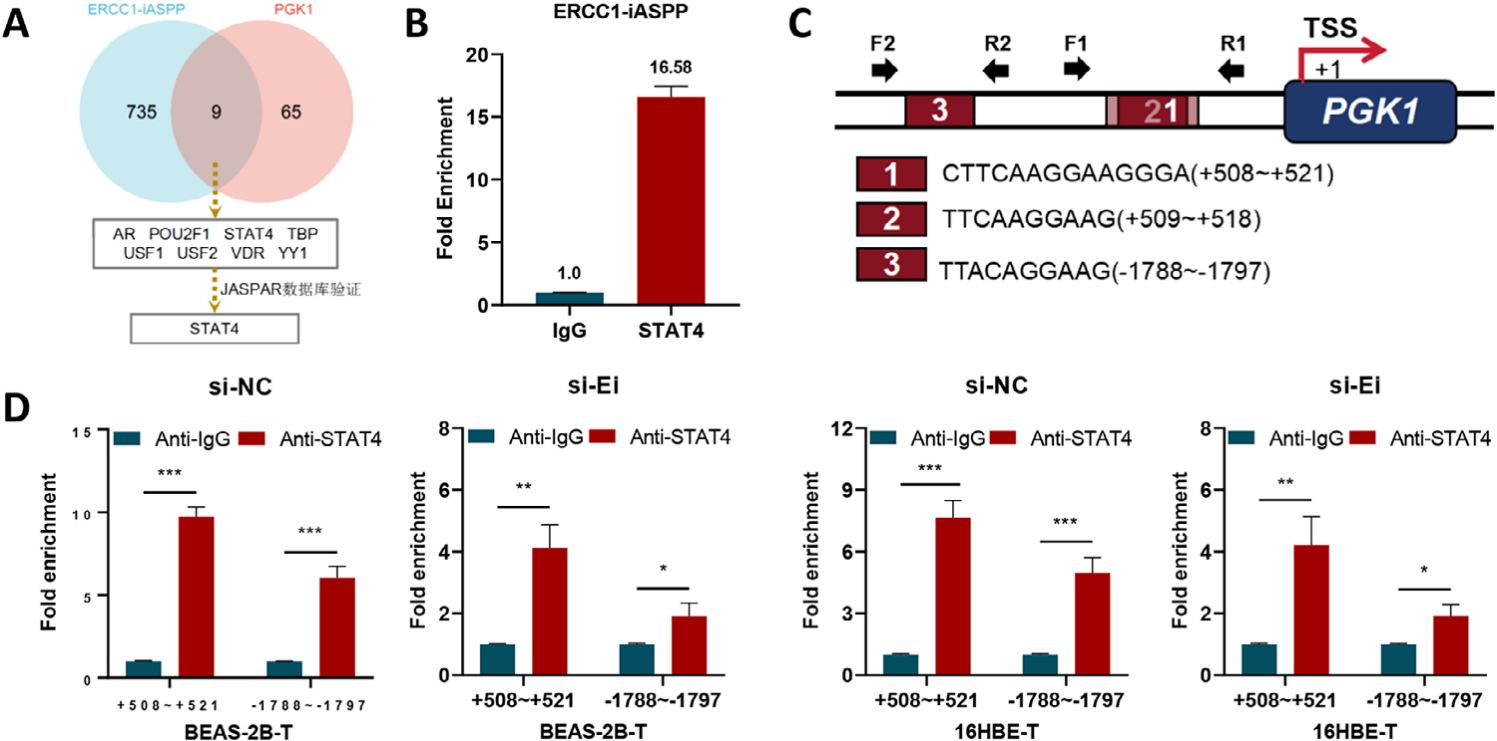
Recruitment of STAT4 by the lnccRNA *ERCC1‐iASPP* in the nucleus enhances *PGK1* transcriptional activity. A,B) Database prediction A) and RIP experiments B) were used to screen for interacting transcription factors. C) Database prediction and schematic of ChIP‐qPCR specific primer design of the 3 highest scoring potential binding sites for STAT4 in the *PGK1* promoter. D) Altered STAT4 binding to *PGK1* promoter sequences in maltransformed cells with altered *ERCC1‐iASPP* expression detected by ChIP‐qPCR. ^*^ *P* < 0.05, ^**^ *P* < 0.01 and ^***^ *P* < 0.001. Data represent the mean ± SD. B, D: n = 3, Student's *t*‐test.

Recent studies have highlighted the critical role of nuclear‐localized lncRNAs in regulating transcriptional programs, often through spatial coordination of transcription factor (TF) complexes.^[^ ^44^ ^]^ The functional plasticity of lncRNAs, governed by their subcellular compartmentalization and mechanistic heterogeneity, suggests that individual molecules may deploy distinct regulatory functions depending on their localization. The present study was undertaken to address the issue of whether nuclear‐enriched *ERCC1‐iASPP* transcriptionally regulates *PGK1* and *CDK1*. The bioinformatic analysis of TF‐binding motifs revealed nine shared TFs between *ERCC1‐iASPP* and *PGK1*, but none with *CDK1* (**Figure** **9**A). Subsequent JASPAR‐based motif refinement and RIP assays identified STAT4 as a direct binding partner of *ERCC1‐iASPP* (Figure 9B).

To resolve the mechanistic role of STAT4 in this regulatory axis, ChIP‐qPCR was performed targeting the three highest‐confidence STAT4 binding motifs in the *PGK1* promoter was performed, as predicted by the Biosignal algorithms (Figure 9C). The results confirmed direct occupancy of STAT4 at these promoter regions. Notably, STAT4 binding to the *PGK1* promoter was significantly reduced in a dose‐dependent manner upon *ERCC1‐iASPP* knockdown (Figure 9D), suggesting that *ERCC1‐iASPP* facilitates the recruitment of STAT4 to chromatin. These findings establish a novel mechanism by which nuclear‐localized *ERCC1‐iASPP* functions as a scaffold for lnccRNA, forming a transcriptional activation complex with STAT4 to directly drive *PGK1* expression.

## 3 Discussion

Chimeric RNAs can originate from two distinct mechanisms: genomic rearrangements at the DNA level or post‐transcriptional events at the RNA level.^[^ ^69^ ^]^ Increasing attention has been directed toward cis‐SAGe—a transcriptional read‐through phenomenon in which RNA polymerase II bypasses normal termination signals, resulting in uninterrupted transcription of neighboring genes located on the same chromosome.^[^ ^70^ ^]^ The resulting transcripts undergo cis‐splicing to generate novel RNA species comprising partial exons and/or introns from both genes. In the present study, we demonstrated that the chimeric RNA *ERCC1‐iASPP* arose from this cis‐splicing mechanism rather than from genomic fusion. Genomic DNA sequencing of the chimeric RNA PCR product confirmed the absence of structural rearrangements or deletions between *ERCC1* and *iASPP*, indicating that their genomic integrity remained intact. Moreover, PCR amplification detected transcriptional products spanning the intergenic region, further supporting a transcriptional rather than genomic origin. To precisely characterize the splicing junctions of *ERCC1‐iASPP*, we designed specific primers targeting the predicted 3' and 5' splice sites and performed RACE. These experiments successfully identified a unique fusion transcript, confirming the existence and sequence composition of the *ERCC1‐iASPP* chimeric RNA.

While previous studies have largely focused on comparing chimeric RNA expression between tumor and adjacent normal tissues, such binary approaches overlook the dynamic transcriptomic remodeling that occurs during stepwise malignant transformation—a hallmark of chemical carcinogenesis. To overcome this limitation, we employed an IARC‐endorsed in vitro cellular malignant transformation model^[^ ^71^ ^]^ using BEAS‐2B and 16HBE cell lines. These were subjected to repeated low‐dose exposures of BPDE, the active metabolite of B[a]P, to mimic chronic environmental carcinogen exposure. Cells with stable knockdown of *ERCC1‐iASPP* exhibited delayed transformation kinetics and reduced tumorigenic potential, while *ERCC1‐iASPP* expression positively correlated with progressive acquisition of malignant phenotypes. These findings highlight its potential as a dynamic biomarker for tobacco‐related lung carcinogenesis.

A comprehensive analysis^[^ ^72^ ^]^ of 297 cis‐SAGe chimeric RNAs previously revealed considerable heterogeneity in their open reading frame (ORF) architecture. While 16% retained full ORFs from both parental genes, the majority (79%) preserved ORF integrity from only one, implying substantial protein‐coding potential among cis‐SAGe products. Additionally, an important criterion is that cytoplasmic RNA is directly associated with ribosomes, the sites of peptide synthesis.^[^ ^73^ ^]^ The essence of encoding involves specific RNA frames being translated into proteins by ribosomes. In line with this, we found that *ERCC1‐iASPP* encoded a functional protein bearing conserved select structural domains from both parental genes, yet displaying novel properties. As a molecular sensor of genotoxic stress, the Ei protein reinforced ERCC1 function, amplifying the DNA repair response to PAH‐induced lesions. Future work will employ interactome mapping and pathway perturbation analyses to delineate the full spectrum of cellular networks regulated by this chimeric protein.

Approximately 75% of the human genome is transcribed, with lncRNAs accounting for ≈98% of these transcripts.^[^ ^74^ ^]^ The biological functions of lncRNAs are highly dependent on their subcellular localization,^[^ ^75^ ^]^ with cytoplasmic lncRNAs often functioning as ceRNAs that sequester miRNAs, thereby relieving miRNA‐mediated repression of target mRNAs. Our findings revealed that cytoplasmic *ERCC1‐iASPP* acted through this ceRNA mechanism by binding to miR‐143‐3p, leading to the upregulation of two key targets, CDK1 and PGK1. This established a previously unrecognized role for miR‐143‐3p in exogenous chemical carcinogenesis, identifying the miR‐143‐3p/CDK1 and miR‐143‐3p/PGK1 axes as critical mediators of B[a]P‐induced lung tumorigenesis. Notably, nuclear‐localized *ERCC1‐iASPP* further amplified this effect by recruiting the transcription factor STAT4 to the *PGK1* promoter, enhancing its transcription and creating a self‐reinforcing regulatory loop that integrated nuclear transcriptional activation with cytoplasmic ceRNA function. This cross‐compartmental positive feedback mechanism provides a plausible explanation for the limited efficacy of therapies targeting single oncogenic pathways and underscores the need for multi‐targeted therapeutic strategies that simultaneously disrupt upstream transcriptional regulation and downstream signaling cascades.

Collectively, our findings demonstrated that *ERCC1‐iASPP* orchestrated PAH‐induced lung carcinogenesis via dual functional modalities. During the early phases of malignant transformation, its coding potential predominated, facilitating DNA repair and cellular survival. As the transformation progressed, its lncRNA‐mediated regulatory functions became increasingly dominant, promoting tumor growth and immune evasion. Despite this functional dichotomy, the precise temporal and quantitative contributions of each modality remain to be fully elucidated. Bifunctional RNAs exhibit spatial compartmentalization of their activities:^[^ ^76^ ^]^ coding functions are executed via ORFs, while non‐coding regions (e.g., 5'/3'UTRs, introns) engage in structural or sequence‐based interactions with RNA‐binding proteins, miRNAs, or chromatin regulators. Importantly, their dual functionality exhibits remarkable plasticity, adapting to cell type, developmental stage, and environmental context.^[^ ^77^ ^]^ Such RNAs are embedded in highly interactive regulatory circuits, where miRNA binding can either trigger translational repression or, conversely, activate gene expression via miRNA titration.

The tumor specificity and unique sequences of chimeric RNA hold great promise for its dual application in precision diagnosis and targeted tumor therapy.^[^ ^78^, ^79^, ^80^, ^81^ ^]^ As demonstrated by Lin et al.,^[^ ^82^ ^]^ the presence of *GOLM1‐NAA35* in saliva‐derived extracellular vesicles serves as a reliable indicator of head and neck squamous cell carcinoma. The tumor preference of *ERCC1‐iASPP* aligns with the tumor‐targeting properties previously reported, indicating its significant potential as a novel non‐invasive biomarker for early tumor diagnosis and monitoring treatment efficacy. Subsequent exploration of its expression in bodily fluids (e.g., blood) has the potential to advance liquid biopsy applications. Excitingly, chimera's unique sequence constitutes an ideal source of neoantigens, laying a solid foundation for the development of precision immunotherapies. A recent study^[^ ^83^ ^]^ on chimeric RNA vaccines has successfully validated the efficacy of this strategy in stimulating anti‐tumor immunity, providing a clear and feasible pathway for translating *ERCC1‐iASPP* into therapeutic vaccines. Furthermore, given its well‐defined oncogenic function, targeted inhibition using technologies such as small‐molecule inhibitors or antisense oligonucleotides represents another highly attractive therapeutic strategy. The chimeric RNA *ERCC1‐iASPP*, first identified in this study, not only reveals a novel mechanism of tumor regulation but also bridges the complete translational pathway from biomarker to innovative therapy, providing robust theoretical support and direction for subsequent clinical translation research.

While the present study has demonstrated the dual coding and non‐coding mechanism of ERCC1‐iASPP in PAHs‐induced lung cancer, there are several limitations that should be kept in mind: 1) The precise molecular mechanisms underlying *ERCC1‐iASPP* formation via cis‐SAGe—including transcriptional read‐through, splice site selection, and RNA processing—are not fully elucidated. Future studies will apply high‐throughput screening and molecular assays to define the upstream regulatory network of the chimera. 2) A specific antibody targeting the ERCC1‐iASPP junctional region is crucial for the direct detection of endogenous protein levels and their functional validation. We prioritize developing such an antibody to enable Western blot and IHC analysis in cell lines and clinical samples, as well as IP‐MS in BPDE‐exposed cells to identify interactors and modifications. 3) Although we provide multiple lines of evidence supporting the oncogenic role of *ERCC1‐iASPP*, direct functional comparisons between the chimera and its parental genes, *ERCC1* and *iASPP*, were not performed due to their low abundance or overlapping phenotypes. Nonetheless, chimera‐specific mechanisms like miR‐143‐3p ceRNA sponging and STAT4‐*PGK1* interaction suggest its biological novelty. The subsequent work will include multi‐omics profiling after specific endogenous knockdown and controlled functional assays to further clarify the unique role of *ERCC1‐iASPP*. 4) For bifunctional RNAs like *ERCC1‐iASPP*, current techniques cannot fully capture dynamic functional switching, comprehensively map interactomes, or precisely delineate coding/non‐coding functional boundaries. Future research should be geared toward integrating high‐resolution spatiotemporal imaging, single‐cell transcriptomics, and advanced bioinformatics, along with RNA‐focused assays (RIP, ribosome profiling, and single‐molecule tracking), to address these aspects at the subcellular level. In conclusion, our current study first identifies and validates the oncogenic role of *ERCC1‐iASPP*, establishing its significance as a novel functional driver in chemical carcinogenesis and thereby laying a solid foundation for deeper mechanistic research.

## 4 Conclusion

**Figure 10:**
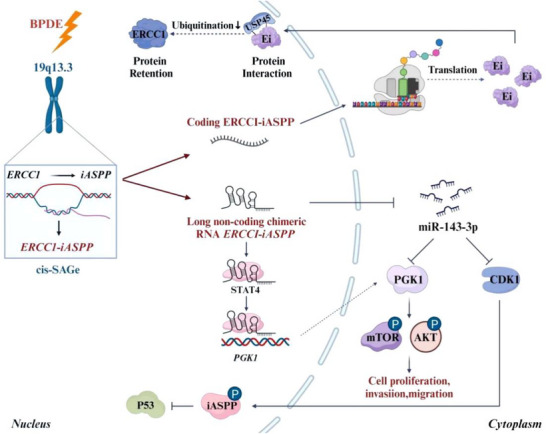
Schematic diagram of chimeric RNA *ERCC1‐iASPP* exerting bifunctional RNA function to regulate the process of malignant cell transformation.

The current study identified a novel chimeric RNA, *ERCC1‐iASPP*, which played a pivotal dual role in the malignant transformation of bronchial epithelial cells upon exposure to PAHs, including the prototypical carcinogen B[a]P. This bifunctional RNA exerted its oncogenic potential via both protein‐coding and non‐coding regulatory mechanisms. As a coding RNA, *ERCC1‐iASPP* encoded a neo‐protein, termed Ei, which stabilized the ERCC1 protein by directly interacting with the deubiquitinating enzyme USP45. This interaction attenuated ERCC1 ubiquitination, thereby enhancing the DNA damage repair capacity of the cell. In its non‐coding role, cytoplasmic *ERCC1‐iASPP* functioned as a ceRNA, sequestering miR‐143‐3p and thereby relieving repression of its target genes *CDK1* and *PGK1*. Upregulation of CDK1 promoted phosphorylation of iASPP, facilitating its nuclear translocation and suppression of p53‐mediated apoptosis. Simultaneously, elevated PGK1 levels stimulated mTOR and AKT phosphorylation, activating oncogenic downstream pathways. Importantly, nuclear‐localized *ERCC1‐iASPP* established a positive transcriptional feedback loop by recruiting STAT4 to the *PGK1* promoter, further enhancing its transcription and sustaining the pro‐tumorigenic signaling axis (**Figure** **10**). This coordinated nuclear–cytoplasmic crosstalk underscores the multifaceted regulatory potential of *ERCC1‐iASPP*.

## 5 Experimental Section

### Clinical Sample Analysis

This study received ethical approval from the Institutional Review Board of China Medical University (Approval No.: [2021]45) and was conducted in compliance with the Declaration of Helsinki principles. Written informed consent was obtained from all participants prior to tissue collection. Paired carcinoma and adjacent non‐tumorous tissues were obtained from 60 histologically confirmed lung cancer patients at Liaoning Cancer Hospital & Institute (Shenyang, China) between February 2021 and February 2022. Histopathological classification and tumor grading were performed according to the 2021 WHO Classification of Thoracic Tumors. Clinico‐pathological data collection included Demographic characteristics (age, sex, smoking history (pack‐years), and alcohol consumption status) and Tumor parameters (histological subtype, maximum tumor diameter, lymphatic metastasis, and TNM staging).

### Cell Culture and BPDE Treatment

Human bronchial epithelial cells (16HBE) and normal lung epithelial cells (BEAS‐2B) were maintained in MEM (HyClone) and DMEM (HyClone), respectively, supplemented with 10% FBS (Biological Industries) at 37 °C/5% CO_2_. A549 and LK_2_ cell lines (National Collection of Authenticated Cell Cultures, China) were cultured in DMEM/F12 and DMEM, respectively. The sublethal BPDE concentrations were determined based on preliminary experiments. To ensure cell viability while inducing malignant transformation, 0.5 and 1 µm BPDE (approximating IC_20_ values) were selected for BEAS‐2B and 16HBE cells. Malignant transformation was induced through repeated low‐dose exposure according to the following protocol: 1) Seeding & Treatment: BEAS‐2B/16HBE cells (1 × 10⁶) were exposed to BPDE for 24 h; 2) Recovery: Washed with fresh medium (10% FBS) and cultured for 7 days; 3) Cyclic Exposure: This 24 h exposure/7‐day recovery cycle was repeated weekly for 4 weeks; 4) Post‐treatment Analysis: Cells underwent serial passaging followed by functional validation: Plate clone formation assay, Soft agar colony formation and Transwell migration assay. A549 and LK_2_ were positive controls; parental BEAS‐2B and 16HBE were negative controls. Successful transformation was confirmed by acquired anchorage‐independent growth and migratory capacity. The malignant transformed cells of 16HBE induced by BPDE at the 40th passage were designated as 16HBE‐T cells, which were also cultured in MEM (HyClone). The malignantly transformed cells of BEAS‐2B induced by BPDE at passage 30 were termed BEAS‐2B‐T cells, which were also cultured in DMEM (HyClone).

### RNA Extraction and RT‐qPCR

Total RNA was isolated from primary lung tissues and cell lines using RNAiso Plus (Takara, Japan), with RNA integrity verified by the values of A260/A280 and A260/A230 between 1.8 and 2.2. Genomic DNA contamination was eliminated through DNase I digestion during RNA purification. First‐strand cDNA synthesis was performed with 1 µg total RNA using the Hifair III 1st Strand cDNA Synthesis SuperMix for qPCR (gDNA digester plus) (Yeasen, China) under strict RNase‐free conditions. RT‐qPCR was performed using Hieff UNICON Universal Blue qPCR SYBR Green Master Mix (Yeasen, China) on a Light Cycler 480 Real‐time PCR System (Roche, USA).

### Rapid Amplification of cDNA Ends and Sanger Sequence

After quality control of the extracted RNA, 3' (Sangon, China) and 5' (Sangon, China) RACE experiments were performed according to the instructions. 1.2% agarose gel (Sangon, China) was cast in 1×TAE buffer, using a microwave‐assisted dissolution protocol, and the amplification products were separated by electrophoresis at 120 V for 30 min to 1 h. The amplified DNA products were analyzed by agarose gel electrophoresis and visualized using a UV analyzer (TANON, China). The DNA sample was then sequenced by Sangon Biotechnology, Shanghai, China.

### Determination of BPDE‐DNA Adducts in Lung Cancer Tissues

Genomic DNA was extracted from human lung cancer tissues using the Universal Genomic DNA Extraction Kit (Takara, Japan). BPDE‐DNA adduct levels were quantified with the BPDE‐DNA Adduct ELISA Kit (Cell Biolabs, USA) according to the manufacturer's protocol. Specifically, DNA samples (4 µg mL^−1^) were coated onto 96‐well plates and incubated with primary anti‐BPDE antibody (1:1000 dilution) for 2 h at 25°C. After three washes with PBS‐T buffer, HRP‐conjugated secondary antibody (1:1000 dilution) was applied for 1 h at 25°C, followed by TMB substrate incubation for 20 min at room temperature. The enzymatic reaction was terminated with 2 m sulfuric acid, and absorbance values were recorded at 450 nm using a microplate reader. The blank control was established with reduced DNA standard, and adduct concentrations were calculated using a standard curve generated from serially diluted BPDE‐DNA standards.

### Fractionation of Nuclear and Cytoplasmic RNA

Subcellular fractionation was performed using the PARIS Kit (Invitrogen, China) following the manufacturer's instructions. Multiple generations of BPDE‐induced malignant transformed cells were isolated, and *ERCC1‐iASPP* expression levels in the nuclear and cytoplasmic fractions were quantified by RT‐qPCR using *MTRNR1* and *U6* as internal controls.

### Cell Transfection

Gene silencing of *ERCC1‐iASPP* was achieved using small interfering RNA (Jima Biotechnology Co., China). siRNA transfection was performed with Lipofectamine 3000 (Invitrogen, China) following the manufacturer's protocol, with functional assays conducted 24 h post‐transfection.

For gain‐of‐function studies, the *ERCC1‐iASPP* overexpression plasmid and corresponding empty vector (Obio Technology Corp., China) were transfected using Lipofectamine 3000. Transfection efficiency was validated by RT‐qPCR and Western blotting.

Stable knockdown cell lines were generated using short hairpin RNA (shRNA) targeting *ERCC1‐iASPP* or a non‐targeting control. For lentiviral transduction, BEAS‐2B/16HBE cells at 30–40% confluence were incubated with viral particles (MOI = 40) and 8 µg mL^−1^ polybrene for 12 h. Stable clones were selected with 2 µg mL^−1^ puromycin (Beyotime, China) for 14 days, followed by maintenance in 1 µg mL^−1^ puromycin containing medium.

### Cell Proliferation Assays

Cell proliferation was assessed with the CellTiter 96 Aqueous One Solution Cell Proliferation Assay (Promega, USA). Following the manufacturer's protocol, transfected EAS‐2B‐T, 16HBE‐T, LK_2_, and A549 cells were plated in 96‐well plates at a density of 2 × 10^3^ cells/well. Cell viability was monitored at 4, 24, 48, 72, and 96 h post‐seeding. At each time point, 20 µL of MTS reagent was added per well and incubated for 2 h at 37°C. Absorbance was measured at 450 nm using a Synergy H1 microplate reader (BioTek, USA) equipped with Gen5 3.04 software.

Clonogenic survival was evaluated in cells using the colony formation assay. Briefly, Cells were seeded in 6‐well plates at 300 cells/well in 2 mL complete growth medium. Plates were incubated at 37 °C in a 5% CO_2_ humidified incubator for 10–20 days (cell line‐dependent). Colonies (>50 cells/colony) were fixed with ice‐cold 100% methanol for 15 min at 4 °C, air‐dried, and stained with 0.5% (w/v) crystal violet in 20% methanol for 30 min at room temperature. After three washes with PBS, stained colonies were quantified using ImageJ, Data represent mean ± SD from triplicate experiments.

For anchorage‐independent growth evaluation, a soft agar colony formation assay was conducted in 24‐well plates. The base layer consisted of 1.2% agar in 2×MEM (for 16HBE‐T) or 2×DMEM (for BEAS‐2B‐T/LK_2_/A549) medium, while the top layer contained 0.6% agar in complete growth medium. Cells (2 × 10^3^ cells/well in 100 µL medium) were embedded in the top agar layer. Plates were maintained in a humidified 5% CO_2_ incubator at 37 °C for 14 days. Colonies were stained with 0.1% iodonitrotetrazolium chloride (INT, 100 mg mL^−1^ in PBS) for 24 h, and visually countable colonies (>50 µm diameter) were quantified using ImageJ. Data represent mean ± SD from triplicate experiments.

### Transwell Migration/Invasion Assays

The Transwell migration/invasion assay was used to evaluate the migration and invasion ability of various transfected malignant and NSCLC cells. Briefly, cells were sequentially digested, centrifuged, resuspended in serum‐free medium, and then counted using a cell counter. After adjusting the cell concentration, 100 µL of cell suspension containing 3–5 × 10^4^ (migrating) or 5–7 × 10^4^ (invading) cells were inoculated into the Transwell chamber (BIOFIL, China) coated (For invasion) or not (For migration) with the Matrigel matrix (Corning, USA) and then placed in a 24‐well plate containing 700 µL of complete medium. After 24 h of incubation at 37°C, the cells were fixed with methanol for 15 min and then stained with 0.5% crystal violet for 30 min. Finally, the number of cells passing through the chambers was counted using ImageJ software.

### RIP Assay

RIP experiments were performed using the Magna RIP Kit (MagnaMedics, USA). Cells were treated at a density of 1 × 10^7^ cells/reaction and lysed for 5 min by the addition of lysis buffer containing protease and RNAase inhibitors. Magnetic beads were conjugated with target antibodies (RPS6, STAT4) or control IgG at room temperature with the addition of the appropriate cell lysate; samples were incubated overnight at 4 °C. Samples were then subjected to RNA purification and protein extraction, and RIP efficiency was verified by protein blotting assay; enrichment of *ERCC1‐iASPP* mRNA was confirmed by RT‐qPCR assay.

### Immunofluorescence

The transfected cell lines were inoculated at the same density into an immunofluorescence culture chamber and cultured using standard protocols. The medium was removed, cells were washed with PBS, and fixed in 4% formaldehyde solution for 15 min. Cells were then permeabilized in 0.1% Triton for 15 min, blocked with 10% serum for 30 min, and incubated overnight at 4 °C using the target antibodies (dilution ratios of each antibody according to the instructions). Cells were stained with secondary antibody (1:150) for 1 h and DAPI for 1–5 min and then imaged under a fluorescence microscope (Olympus, Japan).

### Protein Extraction and Western Blotting

Total protein was extracted using the RIPA Lysis Buffer Kit (Beyotime, China) and quantified by the BCA Protein Detection Kit (TaKaRa, Japan). Western blotting was performed according to the standard method described previously using the following antibodies: anti‐FLAG (Proteintech, 20543‐1‐AP, 1:20000), anti‐ERCC1 (Beyotime, AG1869, 1:1000), anti‐iASPP (Proteintech, 18590‐1‐AP, 1:3000), anti‐Phospho‐Histone H2AX (Beyotime, AF5836, 1:700), anti‐USP45 (Thermo Fisher, IL, PA5‐101946; 1:800), anti‐GAPDH (Proteintech, 60004‐1‐Ig, 1:10000), anti‐Histon H3 (Beyotime, AF0009, 1:1000), anti‐CDK1 (Proteintech, 19532‐1‐AP, 1:5000), anti‐PGK1 (Proteintech, 17811‐1‐AP, 1:10000), anti‐P53 (Proteintech, 10442‐1‐AP, 1:10000), anti‐mTOR (Proteintech, 66888‐1‐Ig, 1:10000), anti‐AKT (Proteintech, 10176‐2‐AP, 1:10000), Anti‐Phospho‐mTOR (Proteintech, 67778‐1‐Ig, 1:5000), Anti‐Phospho‐AKT (Proteintech, 66444‐1‐Ig, 1:3000).

### Co‐Immunoprecipitation

The detection of the protein‐protein interaction between FOSB and p53 in the nucleus using the Nuclear Complex Co‐IP kit (Active Motif, USA), strictly adhering to the manufacturer's protocol. In brief, cells were harvested in ice‐cold PBS/Phosphatase Inhibitors, pelleted by centrifugation at 430 ×g for 5 min at 4 °C, and resuspended in 1× hypotonic buffer. Following 15 min of ice incubation, nuclear fractions were isolated via centrifugation at 14 000 ×g for 30 s. The nuclear pellets were digested with enzymatic Shearing Cocktail in complete digestion buffer for 90 min at 4 °C, followed by centrifugation at 14 000 ×g for 10 min to collect nuclear lysates. For immunoprecipitation, nuclear lysates were incubated overnight at 4 °C with rotation using either Anti‐iASPP antibody (Proteintech, 18590‐1‐AP, 1:50) or Anti‐USP45 antibody (Thermo Fisher, IL, PA5‐101946; 1:100). Immune complexes were subsequently conjugated to Protein G magnetic beads overnight at 4 °C, followed by a 1 h rotational incubation to ensure binding saturation. Wash Buffer to acquire purified protein samples. Finally, the Western Blot was conducted as described above to visualize the potential protein‐protein interaction between Ei and USP45.

### Luciferase Reporter Assay

LK_2_ or BEAS‐2B cells were added to 24‐well plates, reaching 80% confluence the following day. The cells were then co‐transfected with *ERCC1‐iASPP* luciferase and Renilla luciferase reporter plasmids, as well as miR‐143‐3p mimic or control (for LK_2_); miR‐143‐3p inhibitor or control (for BEAS‐2B). Luciferase activity was subsequently assayed using a luciferase reporter gene assay kit (Yeasen, China). The luciferase assay was performed thrice in triplicate.

### Chromatin Immunoprecipitation (ChIP)‐qPCR Assay

The EZ‐Magna ChIP A/G Chromatin Immunoprecipitation Kit (Merck Millipore, USA) was used. Briefly, cells were sequentially cross‐linked with 1% formaldehyde for 10 min, then sufficiently fragmented by sonication to obtain DNA fragments between 200 and 1000 bp in length, and centrifuged at 10 000 × g for 10 min to obtain the supernatant (total lysate). The cell lysate was then incubated with a mixture of anti‐STAT4 specific antibody (proteintech, 13028‐1‐AP, 1:50) and Protein A/G beads on a rotator overnight at 4 °C to induce Protein A/G bead‐antibody/chromatin complexes, whereupon the DNA fragments of interest were extracted from the magnetic beads using microarrays containing Proteinase K elution buffer from the magnetic beads. After the purification process, the collected DNA fragments bound to STAT4 were finally subjected to RT‐qPCR analysis as described above to demonstrate the potential interaction between STAT4 and the PGK1 promoter.

### Statistical Analysis

All quantitative data were presented as mean ± standard deviation (x ± SD). Statistical analyses were performed using SPSS (version 19.0; IBM Corporation, USA) and GraphPad Prism (version 6.0; GraphPad Software Inc., USA). Group comparisons were conducted with either one‐way ANOVA (for multiple groups) followed by Tukey's post hoc test or unpaired Student's *t*‐test (for two‐group comparisons). Bivariate correlations between *ERCC1‐iASPP* and other genes were assessed using Pearson's correlation coefficient. All in vitro assays were conducted independently a minimum of three times, unless stated otherwise. Statistical significance thresholds were defined as ^*^ *P* < 0.05, ^**^ *P* < 0.01, and ^***^ *P* < 0.001.

## Conflict of Interest

The authors declare no conflict of interest.

## Supporting information



Supporting Information

## Data Availability

The data that support the findings of this study are available in the supplementary material of this article.
