# Supplementary material for: A Unique Chimeric RNA: ERCC1‐iASPP Drives Benzo[a]pyrene‐Induced Lung Carcinogenesis via Dual Coding and Non‐Coding Mechanisms
Source: Adv Sci (Weinh). 2025 Nov 26;13(8):e07217. doi: 10.1002/advs.202507217 (PMC12884745; doi:10.1002/advs.202507217)
Supplement: Supplementary file 1 — Supporting Information [file ADVS-13-e07217-s001.docx]

**Supporting Information**

**Table S1** Correlation between *ERCC1-iASPP* expression in lung cancer tissue and basic patient information

| Patients’ information | *ERCC1-iASPP* expression level  Low expression High expression | | *P* value* |
| --- | --- | --- | --- |
| Age |  |  |  |
| ≤ 60 | 15 | 17 | 0.809 |
| ＞60 | 14 | 14 |  |
| Gender |  |  |  |
| Male | 14 | 19 | 0.194 |
| Female | 16 | 11 |  |
| Smoking |  |  |  |
| Yes | 8 | 21 | **0.001** |
| No | 22 | 9 |  |
| Drinking |  |  |  |
| Yes | 8 | 8 | 0.876 |
| No | 21 | 23 |  |
| Diameters(cm) |  |  |  |
| ≤ 3.8 | 16 | 11 | 0.194 |
| > 3.8 | 14 | 19 |  |
| Lymphatic metastasis |  |  |  |
| Yes | 8 | 19 | **0.004** |
| No | 22 | 11 |  |
| TNM stage |  |  |  |
| Ⅰ+Ⅱ | 22 | 12 | **0.009** |
| Ⅲ+Ⅳ | 8 | 18 |  |

Note: This table is a baseline information table + one-way chi-squared analysis for clinical correlation statistical analysis using SPSS statistical software. High/low expression of *ERCC1-iASPP* was determined based on the median of the sample. Pearson 2 test was used as the statistical method. **P* < 0.05 was statistically significant.

| 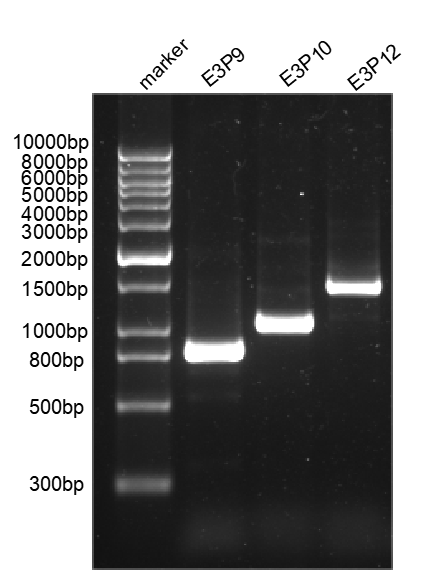 **A**  **B** 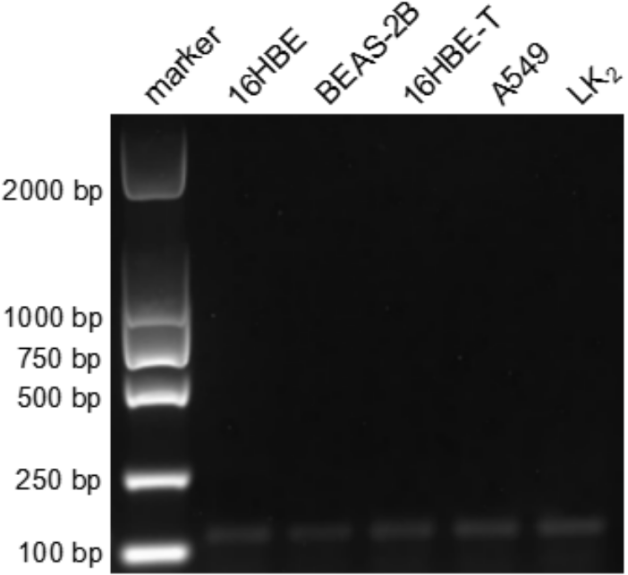 |
| --- |

**Figure S1.** A) Using genomic DNA from *ERCC1-iASPP*-expressing cell lines as template, agarose gel electrophoresis shows that junction-spanning PCR primers failed to amplify the chimeric fragment. B) Long-range PCR amplification of *ERCC1-iASPP* chimeric junction with flanking primers (5'/3') validates expected amplicon size by agarose electrophoresis.

|  0.5  1 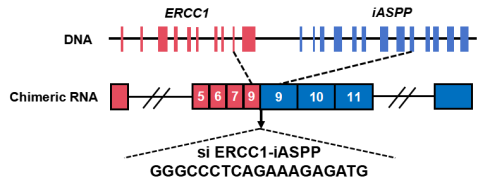 **A**  **B**  **F**  **E**  **D**  **C**  **G** 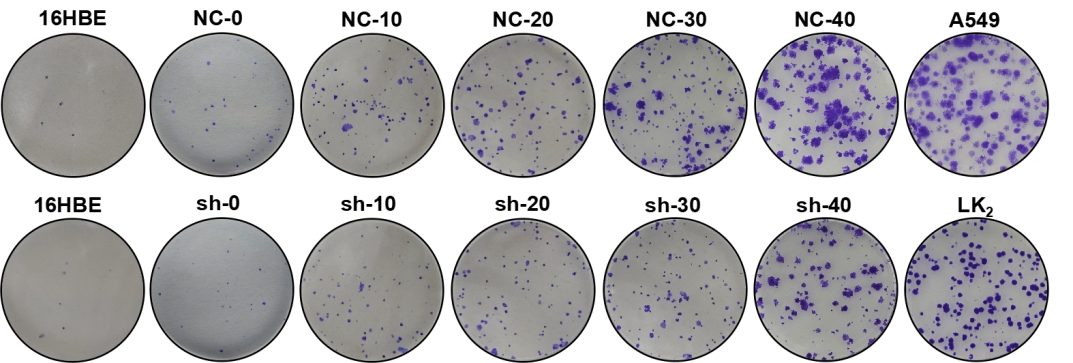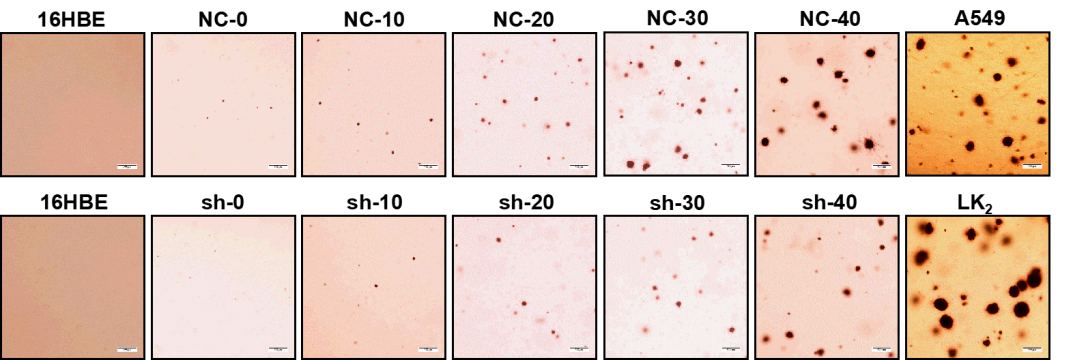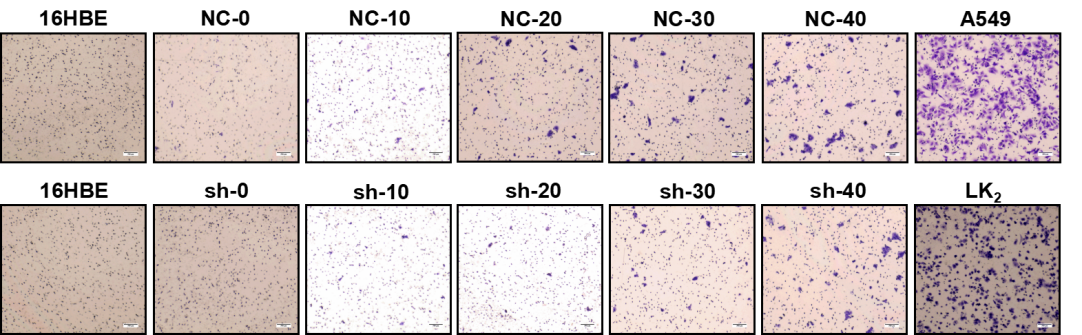 |
| --- |

**Figure S2.** A) RT-qPCR analysis of chimeric *ERCC1-iASPP*, parental *ERCC1*, *iASPP* transcripts in BEAS-2B and 16HBE cells after stable knockdown of *ERCC1-iASPP*. B) MTS screening of BEAS-2B and 16HBE for BPDE exposure dose. C) to E) Stably infected sh-*ERCC1-iASPP* and the negative control 16HBE cells were induced with 1 μM BPDE (24 h each time, once a week for 4 consecutive weeks) respectively, and the plate cloning, soft agar clone formation, and Transwell migration assay were performed to detect the malignancy degree of cells in generation 0, 10, 20, 30 and 40. F) Changes in mRNA expression levels of chimeric RNA and parental genes with the number of generations of transformed cells during BPDE-induced malignant transformation. G) Schematic design of siRNA targeting *ERCC1-iASPP* and RT-qPCR detection of *ERCC1-iASPP* and parental *ERCC1*, *iASPP* changes in mRNA expression after transient knockdown of *ERCC1-iASPP*. ****P* < 0.05, ***P* < 0.01 and ****P* < 0.001. ns: differences were not statistically significant. Scale bar is 500 μm. Data represent the mean ± SD. A, C-E, G: n = 3,** Student’s t test.

| 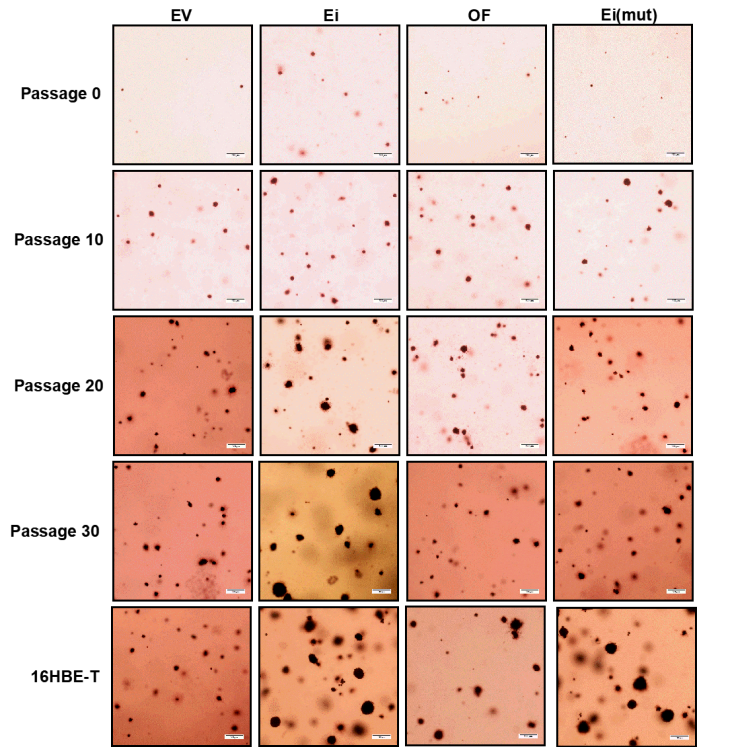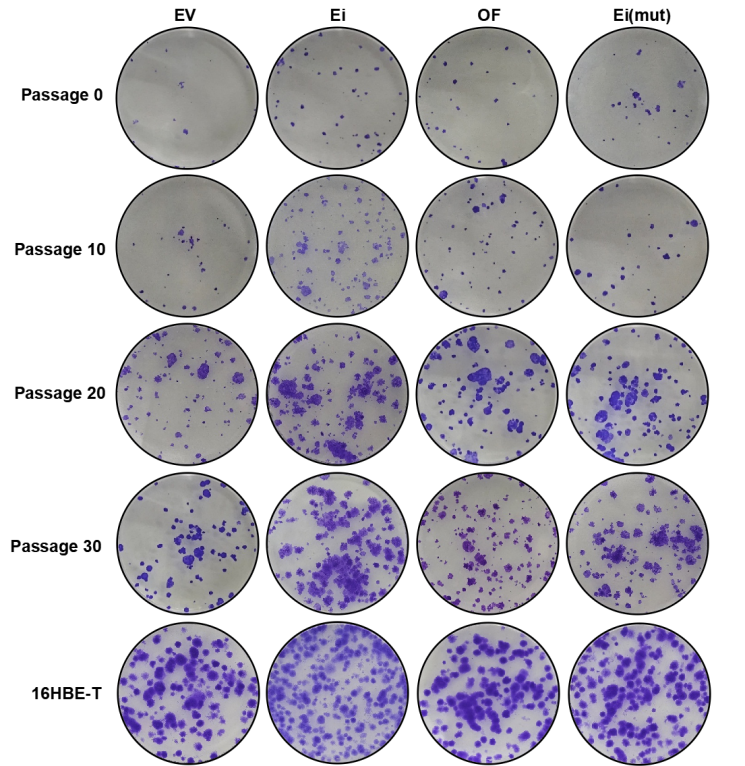 **A**  **B**  **C**  **D** 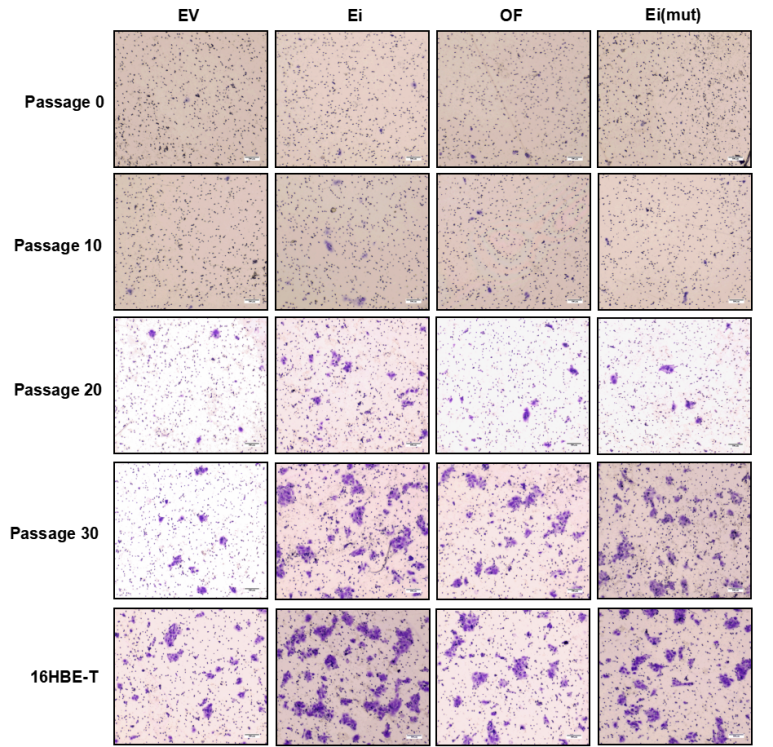 **E** |
| --- |

**Figure S3. A) RT-qPCR analysis of *ERCC1-iASPP* in nuclear and cytoplasm extracts, with *U6* used as a nucleus location reference and *MTRNR* as a cytoplasm location reference. With the enhancement of cell malignancy, *ERCC1-iASPP* mRNA gradually shifted from nuclear distribution to cytoplasmic. B) RT-qPCR was performed to detect *ERCC1-iASPP* mRNA expression levels after BEAS-2B and 16HBE cells were transfected with plasmids representing different attributes of *ERCC1-iASPP*. C) to E) Representative results of plate colony formation, soft agar colony formation, and Transwell migration assays. Effect of plasmids with different properties of *ERCC1-iASPP* on the anchorage-independent growth ability of malignant-transformed cells at each stage. **P* < 0.05, ***P* < 0.01 and ****P* < 0.001. ns: differences were not statistically significant. Scale bar is 500 μm. Data represent the mean ± SD. B-E: n = 3, Kruskal-Wallis test.**

| 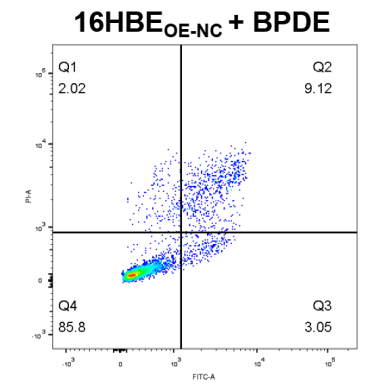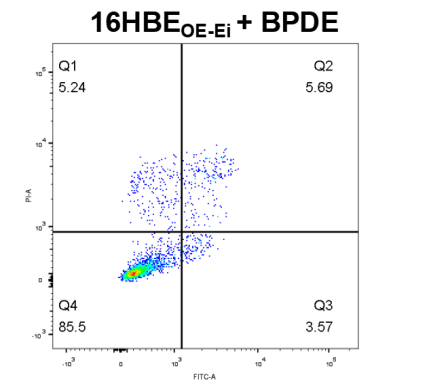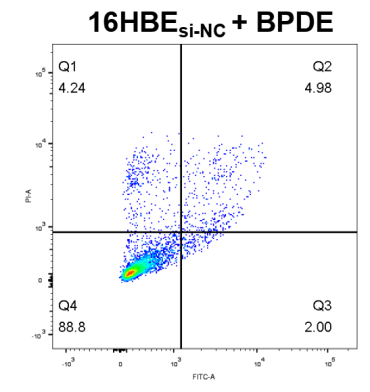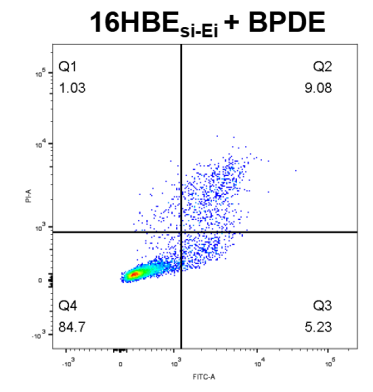 **D**  **G**  **F**  **E**  **A**  **B**  **C** 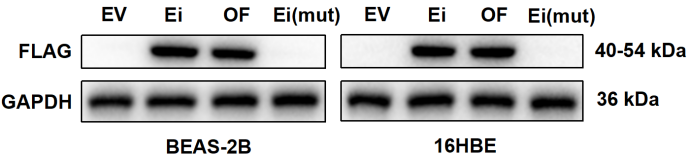 |
| --- |

**Figure S4. A) RT-qPCR analysis of chimeric *ERCC1-iASPP*, parental *ERCC1*, *iASPP* transcripts in BEAS-2B and 16HBE cells after overexpression of *ERCC1-iASPP*. B) Changes in Ei protein levels in BEAS-2B, 16HBE transfected cells expressing different types of plasmids detected by Western blot. C) Quantified ERCC1-iASPP’s protein expression efficiency using FLAG-tagged full-length chimera constructs. D) to E) Flow cytometry D) and MTS assay E) were performed to detect the effects of *ERCC1-iASPP* knockdown on BPDE-induced apoptosis and cell activity in 16HBE cells. F) to G) The effects of *ERCC1-iASPP* overexpression on BPDE-induced apoptosis and cell activity in 16HBE cells were determined by flow cytometry F) and MTS assay G). **P* < 0.05, ***P* < 0.01 and ****P* < 0.001. ns: differences were not statistically significant. Data represent the mean ± SD. A, C-F: n = 3, Student’s t test.**

| 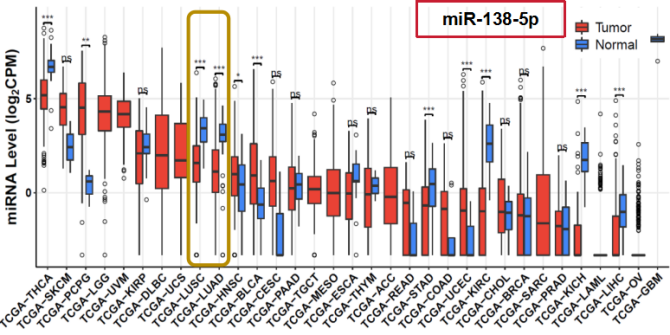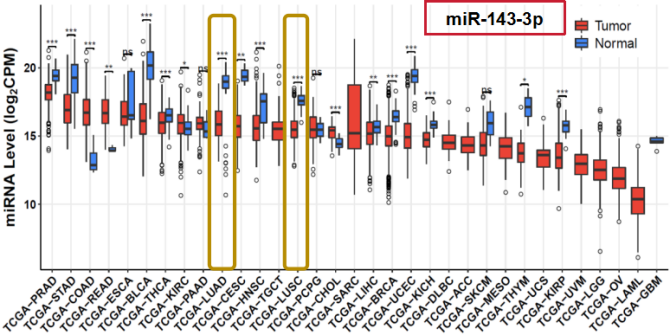 **A**  **E**  **D**  **C**  **B**  **F** |
| --- |

**Figure S5. A) Analysis of candidate microRNA expression in pan-cancer, of which the data were obtained from the TCGA database. B) miR-143-3p expression in various cell lines, in which malignant-transformed cells and lung cancer cells showed lower expression levels. C) to D) RT-qPCR was performed to detect changes in miR-143-3p expression after transfection with miR-143-3p mimic or inhibitor in each cell line. E) to F) RT-qPCR was performed to detect changes in *CDK1*, *PGK1* mRNA expression in cells transfected with miR-143-3p alone or co-transfected with *ERCC1-iASPP*. **P* < 0.05, ***P* < 0.01 and ****P* < 0.001. ns: differences were not statistically significant. Data represent the mean ± SD. B-D: n = 3, Student’s t test. E, F: n = 3, Kruskal-Wallis test.**

| 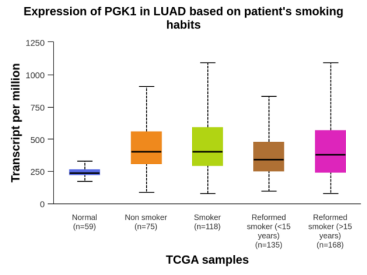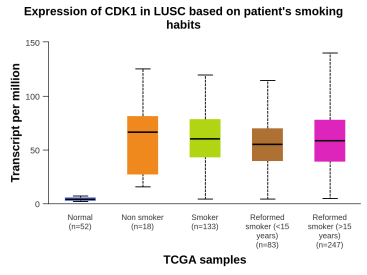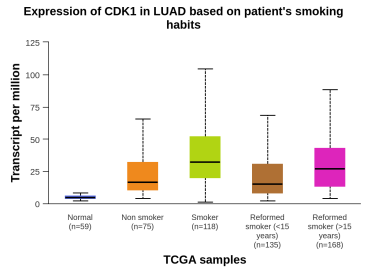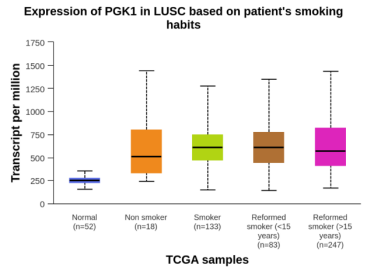 **B**  **E**  **C**  **D** 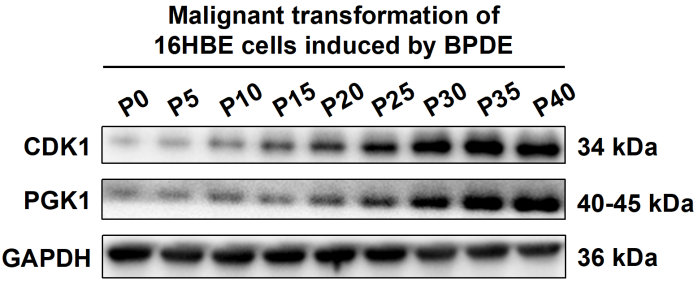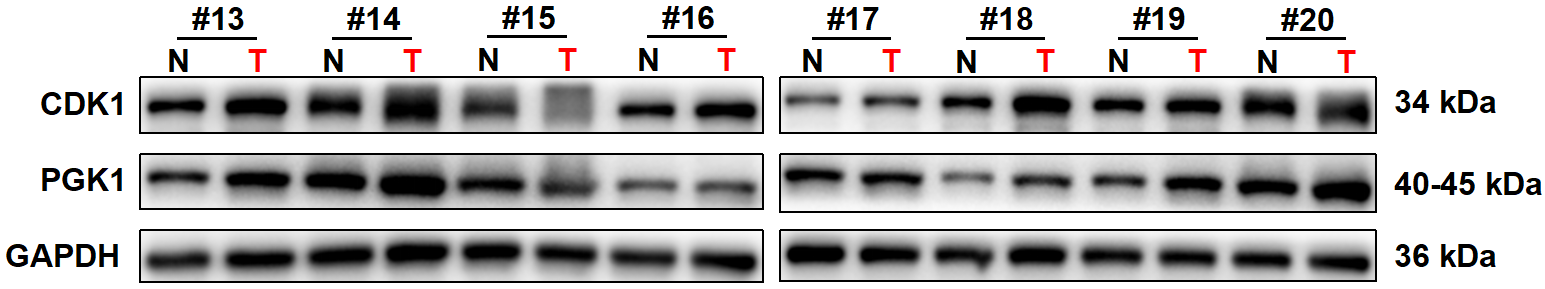 **A** |
| --- |

**Figure S6.** A) Differential expression of the target genes, CDK1 and PGK1 proteins, in lung cancers and paired paracancerous tissues. **B) The correlation between CDK1, PGK1 protein and *ERCC1-iASPP* mRNA in lung cancers with paired adjacent non-tumorous tissues. C) RT-qPCR detection of *CDK1* and *PGK1* mRNA expression altered by miR-143-3p mimic after overexpression of *ERCC1-iASPP*. D) The association between CDK1, PGK1 and the number of pack-years of smoking in lung cancer patients, of which the data were obtained from the TCGA database. E) CDK1, PGK1 mRNA and protein expression during BPDE-induced malignant transformation of 16HBE cells. **P* < 0.05, ***P* < 0.01 and ****P* < 0.001. ns: differences were not statistically significant. Data represent the mean ± SD. A: Paired Samples t-test. B: n = 3, Kruskal-Wallis test.**
